# Identification of ubiquitin-specific protease 32 as an oncogene in glioblastoma and the underlying mechanisms

**DOI:** 10.1038/s41598-022-09497-y

**Published:** 2022-04-19

**Authors:** Sifang Chen, Xi Chen, Zhangyu Li, Jianyao Mao, Weichao Jiang, Zhi Zhu, Yukui Li, Zhengye Jiang, Wenpeng Zhao, Guowei Tan, Zhanxiang Wang

**Affiliations:** 1 Department of Neurosurgery, The First Affiliated Hospital of Xiamen University, No. 55 Zhenghai Road, Siming District, Xiamen, 361000 Fujian China; 2 Department of Neurosurgery, Heze Municipal Hospital, Heze, 274000 Shandong China; 3 Xiamen Key Laboratory of Brain Center, The First Affiliated Hospital of Xiamen University, Xiamen, 361000 Fujian China; 4 Department of Neuroscience, Institute of Neurosurgery, School of Medicine, Xiamen University, Xiamen, 361000 Fujian China

**Keywords:** Cancer, Neuroscience

## Abstract

Glioblastoma (GBM) patients present poor prognosis. Deubiquitination by deubiquitinating enzymes (DUBs) is a critical process in cancer progression. Ubiquitin-specific proteases (USPs) constitute the largest sub-family of DUBs. Evaluate the role of USP32 in GBM progression and provide a potential target for GBM treatment. Clinical significance of USP32 was investigated using Gene Expression Omnibus databases. Effects of USP32 on cell growth and metastasis were studied in vitro and in vivo. Differentially expressive genes between USP32-knockdown U-87 MG cells and negative control cells were detected using RNA sequencing and used for Gene Ontology and Kyoto Encyclopedia of Genes and Genomic pathway enrichment analyses. Finally, RT-qPCR was used to validate the divergent expression of genes involved in the enriched pathways. USP32 was upregulated in GBM patients, being correlated to poor prognosis. USP32 downregulation inhibited cell growth and metastasis in vitro. Furthermore, USP32 knockdown inhibited tumorigenesis in vivo. In addition, UPS32 was identified as a crucial regulator in different pathways including cell cycle, cellular senescence, DNA replication, base excision repair, and mismatch repair pathways. USP32 acts as an oncogene in GBM through regulating several biological processes/pathways. It could be a potential target for GBM treatment.

## Introduction

Glioblastoma (GBM), the most aggressive type of glioma, are characterized by cellular heterogeneity, a diffusive infiltration of tumor cells, and the presence of glioma stem-like cells capable of significantly expanding and generating new tumors. GBM patients have an average overall survival time of barely more than 15 months^1–4^. Understanding the molecular mechanisms driving malignancy helps in the development of agents specifically targeting the tumor cells or tumor microenvironment^5^. Despite improvements in therapeutic strategies, GBM remains a clinical challenge. There is an urgent need to find novel regulators or targets for exploring highly bioactive and brain-penetrating targeted therapies.

Ubiquitination is an ATP-dependent cascade process that ligates ubiquitin to a substrate protein, catalyzed by the E1, E2, and E3 three-enzyme cascade^6^. The ubiquitinated proteins can be specially recognized by the 26S proteasome, and this leads to the proteasomal degradation of these proteins, regulating different cell processes. Many studies have revealed that the ubiquitin–proteasome system is involved in the progression of GBM^7–10^. A siRNA screening analysis showed that 22% of GBM-survival-relevant genes were components of the 20S and 26S proteasome subunits^11^. Epidermal growth factor receptor (EGFR) amplification and mutations are commonly observed in GBM; EGFR stability and downstream signaling are subject to the ubiquitin regulatory network^10^. Homologous to E6-AP Carboxyl Terminus (HECT) E3 ligase Smurf2 suppresses TGF-β signaling by targeting TbR-I for proteasomal degradation, promoting the progression of GBM^12^. HECT E3 ligase HERC3 play an essential role in autophagy-induced EMT, resulting in the chemoresistance of GBM^13^. In GBM, the p53 and c-Myc levels are regulated by E3 ligases MDM2 and TRIM3, respectively^14,15^. The ubiquitination is often antagonized by deubiquitinating enzymes (DUBs) which remove the ubiquitin chains from ubiquitinated proteins^16^. More than 100 DUBs have been identified and categorized into 8 subfamilies, of which ubiquitin-specific proteases (USPs) constitute the largest one. DUBs not only function to reverse ubiquitination but are also involved in protein trafficking, chromatin remodeling, cell cycle regulation, base excision repair, mismatch repair, and signaling pathway modulations, which are tightly associated with the development of cancer^17,18^. DUBs have emerged as attractive targets for targeted therapy in cancer^19,20^ after the clinical success of proteasomal inhibitors, such as bortezomib, ixazomib, and carfilzomib. NSC 144303 (G5) and NSC 632839 (F6), two DUBs inhibitors, are under pre-clinical trial^6^. Several DUBs were reported to regulate multiple cellular processes such as apoptosis, proliferation, and stemness in GBM^7,21^.

USP32 is a member of USPs discovered in recent years. Previous studies had shown that USP32 is highly expressed in breast cancer and promotes the growth of breast tumor cells^22^. Hu et al. revealed that USP32 knockdown suppresses cellular proliferation and cell metastasis in small cell lung cancer^23^. The study of Dou et al. reported that high expression of USP32 is significantly associated with high T stage and poor prognosis in gastric cancer patients^24^. USP32 was also identified as an oncogene in epithelial ovarian^25^. However, USP32 is still rarely reported in malignant disease processes, especially in GBM. In this study, USP32 was knocked down in GBM cells, evaluating the effect of this enzyme on cell growth and metastasis. Tumor xenograft experiments in nude mice were also performed to validate the role of USP32 in GBM development. Transcriptional sequencing identified the differential expressed genes between stably USP32-knockdown U-87 MG cells and negative control cells. Functional annotations, including Gene Ontology (GO) and Kyoto Encyclopedia of Genes and Genomes (KEGG) pathway enrichment analyses, were used to uncover the underlying mechanism of USP32 on promoting the progression of GBM. Our study may provide a potential target for GBM treatment.

## Methods

### Cell lines and cell culture

293T, SVG p12, U-87 MG, U-118 MG, U-251 MG, T98G, and A172 cells were purchased from Xiamen Immocell Biotechnology Co., Ltd (Xiamen, Fujian, China). All cells were maintained using Dulbecco's modified Eagle's medium (DMEM, IMMOCELL) containing 15% fetal bovine serum (FBS, Gibco) at 37 °C in an incubator with an atmosphere containing 5% CO_2_ and 21% O_2_.

### High-content screening

Small interfering RNAs (siRNAs) targeting USPs were obtained from GenePharma (Shanghai, China). Table 1 describes these siRNAs. U-87 MG and U-251 MG cells were seeded into 96-well plates at a density of 1 × 10^4^ per well. Lipofectamine RNAiMAX (Life Technology, Carlsbad, CA, USA) was used for siRNA transfection at 37 °C. After a 36 h of transfection, the cells were incubated with 10 μL/well CCK-8 solution (Yeasen, Shanghai, China). The cell viability was evaluated 2 h later by detecting the optical density at 450 nm (OD_450_) using a microplate reader (Molecular Devices, San Francisco, CA, USA).

**Table 1:** Sequences of siRNAs targeting USPs.

siRNAs	Sense (5′–3′)	Antisense (5′–3′)
siUSP1-1780	GGUUAAAGUCUGCAACUAAUU	UUAGUUGCAGACUUUAACCUU
siUSP1-1501	GCAUAGAGAUGGACAGUAUUU	AUACUGUCCAUCUCUAUGCUU
siUSP2-1339	GCGCUUUGUUGGCUAUAAUUU	AUUAUAGCCAACAAAGCGCUU
siUSP2-1921	CCUGUACGCUGUGUCCAAUUU	AUUGGACACAGCGUACAGGUU
siUSP3-1109	GGGACAGAAUCUAGAAAGUUU	ACUUUCUAGAUUCUGUCCCUU
siUSP3-1569	GCUGGUUCCACUUCAAUGAUU	UCAUUGAAGUGGAACCAGCUU
siUSP4-377	GCGUGGAAUAAACUACUAAUU	UUAGUAGUUUAUUCCACGCUU
siUSP4-860	GCAAAUGGUGAUAGCACUAUU	UAGUGCUAUCACCAUUUGCUU
siUSP5-822	GGAGCUGACGUGUACUCAUUU	AUGAGUACACGUCAGCUCCUU
siUSP5-1265	GCCAGAACAGAAGGAAGUUUU	AACUUCCUUCUGUUCUGGCUU
siUSP6-2274	GGAAGGACAUACUUAUGAAUU	UUCAUAAGUAUGUCCUUCCUU
siUSP6-2494	GCACAGUAGCAAACUCAUAUU	UAUGAGUUUGCUACUGUGCUU
siUSP7-2625	GUGGUUACGUUAUCAAAUAUU	UAUUUGAUAACGUAACCACUU
siUSP7-603	GCAGUGCUGAAGAUAAUAAUU	UUAUUAUCUUCAGCACUGCUU
siUSP8-873	CCAAAGAGAAAGGAGCAAUUU	AUUGCUCCUUUCUCUUUGGUU
siUSP8-3569	GCAAGACAACGGUGGUUUAUU	UAAACCACCGUUGUCUUGCUU
siUSP9X-7849	GGGCAAUGGAGAUCUUAAAUU	UUUAAGAUCUCCAUUGCCCUU
siUSP9X-2333	CCCGCACUGAAACAAAUUAUU	UAAUUUGUUUCAGUGCGGGUU
siUSP9Y-2805	CCUUGCAACCUACAUGAAUUU	AUUCAUGUAGGUUGCAAGGUU
siUSP9Y-8135	GCAGUUGUCCUGUUGCUUAUU	UAAGCAACAGGACAACUGCUU
siUSP10-630	GCUUUGGAUGGAAGUUCUAUU	UAGAACUUCCAUCCAAAGCUU
siUSP10-1175	GCACACCACGGAAAGCAUAUU	UAUGCUUUCCGUGGUGUGCUU
siUSP11-1415	GCAAUGUAUCUGUGACCUUUU	AAGGUCACAGAUACAUUGCUU
siUSP11-2088	CCUCCUGGACAAUUGCCUUUU	AAGGCAAUUGUCCAGGAGGUU
siUSP12-314	CUUCGGCAUUAGAGAAAGAUU	UCUUUCUCUAAUGCCGAAGUU
siUSP12-648	CCUACUAAAUACAAUUGCUUU	AGCAAUUGUAUUUAGUAGGUU
siUSP13-1685	CGUGCCAAGAUACCAUUUAUU	UAAAUGGUAUCUUGGCACGUU
siUSP13-2004	GCCUGAUGAACCAAUUGAUUU	AUCAAUUGGUUCAUCAGGCUU
siUSP14-655	GCUUCAGCGCAGUAUAUUAUU	UAAUAUACUGCGCUGAAGCUU
siUSP14-1632	GCAUAUCGCUUACGUUCUAUU	UAGAACGUAAGCGAUAUGCUU
siUSP15-249	GGAACACCUUAUUGAUGAAUU	UUCAUCAAUAAGGUGUUCCUU
siUSP15-1150	GCAGAUGGAAGGCCAGAUAUU	UAUCUGGCCUUCCAUCUGCUU
siUSP16-331	GGAAUGGAAUAUCUGCCAAUU	UUGGCAGAUAUUCCAUUCCUU
siUSP16-469	GCAUGCCUUGAAGCACUAUUU	AUAGUGCUUCAAGGCAUGCUU
siUSP17-1431	CCAUCAUCCUGAACAGCAAUU	UUGCUGUUCAGGAUGAUGGUU
siUSP17-1538	GGAGAUCCAAAGGGAAGAAUU	UUCUUCCCUUUGGAUCUCCUU
siUSP18-527	GCUGCCUUAACUCCUUGAUUU	AUCAAGGAGUUAAGGCAGCUU
siUSP18-1418	CUGCAUAUCUUCUGGUUUAUU	UAAACCAGAAGAUAUGCAGUU
siUSP19-2439	GCAUUCAGAACAAGCCCUAUU	UAGGGCUUGUUCUGAAUGCUU
siUSP19-2518	GCGGCACAAGAUGAGGAAUUU	AUUCCUCAUCUUGUGCCGCUU
siUSP20-249	CCAUAGGAGAGGUGACCAAUU	UUGGUCACCUCUCCUAUGGUU
siUSP20-1041	GCCCAUCAGAAGAUGAGUUUU	AACUCAUCUUCUGAUGGGCUU
siUSP21-641	CCAACUUAGCCCGUUCCAAUU	UUGGAACGGGCUAAGUUGGUU
siUSP21-1353	GCUAGAAGAACCUGAGUUAUU	UAACUCAGGUUCUUCUAGCUU
siUSP22-1367	GCUACCAGGAGUCCACAAAUU	UUUGUGGACUCCUGGUAGCUU
siUSP22-695	GGAGAAAGAUCACCUCGAAUU	UUCGAGGUGAUCUUUCUCCUU
siUSP24-611	GGAAUUGAAUUCCCUACAAUU	UUGUAGGGAAUUCAAUUCCUU
siUSP24-719	GCAUCUACCUACCUAGCAAUU	UUGCUAGGUAGGUAGAUGCUU
siUSP25-897	GCCAAAGAACCCUAUGGUAUU	UACCAUAGGGUUCUUUGGCUU
siUSP25-1128	GCCGGUAUUAACAUUUGAAUU	UUCAAAUGUUAAUACCGGCUU
siUSP26-1600	CCUUAUUGUUCACCUCAAAUU	UUUGAGGUGAACAAUAAGGUU
siUSP26-2426	GGUUCCAAUAAGAAUCCAAUU	UUGGAUUCUUAUUGGAACCUU
siUSP27-1398	GGCGCAAGAUCACUACAUAUU	UAUGUAGUGAUCUUGCGCCUU
siUSP27-855	CUCCUCAUGUGCCCUAUAAUU	UUAUAGGGCACAUGAGGAGUU
siUSP28-1836	GGGCCUAUAUCUAUAAUCAUU	UGAUUAUAGAUAUAGGCCCUU
siUSP28-841	GCAUUCCAGCUAGCUGUUAUU	UAACAGCUAGCUGGAAUGCUU
siUSP29-983	CCCAUCAAGUUUAGAGGAUUU	AUCCUCUAAACUUGAUGGGUU
siUSP29-1922	GGUGAAGAAUAACGAGCAAUU	UUGCUCGUUAUUCUUCACCUU
siUSP30-176	CCGUCAGAUAUAAAGUCAUUU	AUGACUUUAUAUCUGACGGUU
siUSP30-521	GCUGCUUGUUGGAUGUCUUUU	AAGACAUCCAACAAGCAGCUU
siUSP31-954	GCCUCUCUAUGUCACUGUAUU	UACAGUGACAUAGAGAGGCUU
siUSP31-3922	GCUCGCAAAUCCAAGUCUUUU	AAGACUUGGAUUUGCGAGCUU
siUSP32-2261	GCGCAUUAAAGAGGAAGAUUU	AUCUUCCUCUUUAAUGCGCUU
siUSP32-386	GACCUGUGGACUCUCAUAUUU	AUAUGAGAGUCCACAGGUCUU
siUSP33-829	CCCAGUAAUACAACAUUAAUU	UUAAUGUUGUAUUACUGGGUU
siUSP33-597	GGAGAAUAGAUGUUCAUAUUU	AUAUGAACAUCUAUUCUCCUU
siUSP34-1228	GCGACUGAGUACUCAACAUUU	AUGUUGAGUACUCAGUCGCUU
siUSP34-3023	CCUGAUCAUUUCAGGUUAAUU	UUAACCUGAAAUGAUCAGGUU
siUSP35-3188	CCCUGCACAAGGACUUGAUUU	AUCAAGUCCUUGUGCAGGGUU
siUSP35-1916	GCUCGGAGUAUCUGAAGUAUU	UACUUCAGAUACUCCGAGCUU
siUSP36-741	CCAACUACCUGCUCUCCAAUU	UUGGAGAGCAGGUAGUUGGUU
siUSP36-474	GCAAAUAUGUGUUGCUCAAUU	UUGAGCAACACAUAUUUGCUU
siUSP37-555	CCAAGGAUAUUUCAGCUAAUU	UUAGCUGAAAUAUCCUUGGUU
siUSP37-2235	GCACAUAUGGCAAUUUCUAUU	UAGAAAUUGCCAUAUGUGCUU
siUSP38-3501	GGUAAGUUGGAAAUACAAGUU	CUUGUAUUUCCAACUUACCUU
siUSP38-1047	GGUUCGAACGAUAGGCCAUUU	AUGGCCUAUCGUUCGAACCUU
siUSP39-958	GGAACCCUCGAAAUUUCAAUU	UUGAAAUUUCGAGGGUUCCUU
siUSP39-1375	GCAUCACUGAGAAGGAAUAUU	UAUUCCUUCUCAGUGAUGCUU
siUSP40 -721	GCAGCAAAGUCGGCCAAAUUU	AUUUGGCCGACUUUGCUGCUU
siUSP40 -1212	GCUCCAUUCUCAGAUAUUUUU	AAAUAUCUGAGAAUGGAGCUU
siUSP42-459	GCUCCAGAAUUUGGGCAAUUU	AUUGCCCAAAUUCUGGAGCUU
siUSP42-750	GCAGAAAGCAUGCUUGAAUUU	AUUCAAGCAUGCUUUCUGCUU
siUSP43-813	GCCACUUUCAAGCACAAUAUU	UAUUGUGCUUGAAAGUGGCUU
siUSP43-2196	GGGCUUAUAUCCUGUUCUAUU	UAGAACAGGAUAUAAGCCCUU
siUSP44-653	GGGUACAGGUGAUGAUUCUUU	AGAAUCAUCACCUGUACCCUU
siUSP44-1553	CGCUCAGGAAUUUCUUUGUUU	ACAAAGAAAUUCCUGAGCGUU
siUSP45-1377	GGCACCUCGAUUUAAAGAUUU	AUCUUUAAAUCGAGGUGCCUU
siUSP45-753	GCAGCUAGUACUUACUUCUUU	AGAAGUAAGUACUAGCUGCUU
siUSP46-204	GGUCCAGAGCAGUUUCCAAUU	UUGGAAACUGCUCUGGACCUU
siUSP46-426	CCACCAAAGAAGUUCAUUUUU	AAAUGAACUUCUUUGGUGGUU
siUSP47-2463	GCUGUCGCCUUGUUAAAUAUU	UAUUUAACAAGGCGACAGCUU
siUSP47-3757	CCAGCAAUCAAGAGUUUGAUU	UCAAACUCUUGAUUGCUGGUU
siUSP48-676	GCAUCUCCAGUACUUGUUUUU	AAACAAGUACUGGAGAUGCUU
siUSP48-871	GCAGUUCUGUGGAGAAUAUUU	AUAUUCUCCACAGAACUGCUU
siUSP49-1925	GGGUCCAUGUCGUCUUUGAUU	UCAAAGACGACAUGGACCCUU
siUSP49-1825	GAAGCUAGAAAGCAGUUAAUU	UUAACUGCUUUCUAGCUUCUU
siUSP50-571	GCUCAGGAAUUCUUGAUUUUU	AAAUCAAGAAUUCCUGAGCUU
siUSP50-692	CCACUGAGACAUCCAUCAUUU	AUGAUGGAUGUCUCAGUGGUU
siUSP51-895	CCAUUUAGCUGUAGACCUUUU	AAGGUCUACAGCUAAAUGGUU
siUSP51-1225	CCAUAUUCCUCUACUGAAAUU	UUUCAGUAGAGGAAUAUGGUU
siUSP52-896	GCUGCAGAAUCACAUACUAUU	UAGUAUGUGAUUCUGCAGCUU
siUSP52-1247	GCGCUUCAUUCCUACAUAUUU	AUAUGUAGGAAUGAAGCGCUU
siUSP53-3296	GAGCCAACAUCACUUAGAAUU	UUCUAAGUGAUGUUGGCUCUU
siUSP53-1605	GUGCGGUACAUUUCUACAAUU	UUGUAGAAAUGUACCGCACUU
siUSP54-3654	GCUGCCUAAUGGUGAAACUUU	AGUUUCACCAUUAGGCAGCUU
siUSP54-2546	GAGCCCUAGUCGAUAAGAAUU	UUCUUAUCGACUAGGGCUCUU
siRNA NC	CUCCGAACGUGUCACGUU	CGUGACACGUUCGGAGUU

### Data mining from Gene Expression Omnibus (GEO) databases

Dataset GSE59612 (normal 17, GBM 39) was downloaded from the GEO website (https://www.ncbi.nlm.nih.gov/geo/) to evaluate the differential expression of USP32 between normal tissues and tumor tissues. The mRNA expression data and survival information of GBM patients in datasets GSE74187 (GBM 60) and GSE83300 (GBM 50) were also downloaded. The association between USP32 expression level and prognosis was analyzed. GBM patients were divided into two groups (high and low) using the optimal cut-off value of USP32 expression level, which was determined using the surv_cutpoint function of the R package survminer via RStudio software (version 2021.09.0 + 351, https://www.rstudio.com/).

### RT-qPCR

Total RNA from U-87 MG was obtained using an RNA Extraction Kit (Vazyme, Nanjing, Jiangsu, China) and reverse transcribed into cDNA using HiScript II Reverse Transcriptase (Vazyme). One hundred nanogram of cDNA was used for qPCR per well. qPCR was performed using a Bio-Rad CFX96 system (Bio-Rad Laboratories, Hercules, CA, USA) with an AceQ qPCR SYBR Green Master Mix Kit (Vazyme). The thermocycling condition was 96 °C for 5 min, followed by 96 °C for 15 s, 60 °C for 25 s, and 72 °C for 20 s, for 45 cycles. The 2^−ΔΔCt^ method was used to calculate the relative mRNA levels, which was calibrated to 18S RNA. The primers for RT-qPCR are shown in Table 2.

**Table 2:** Primers for RT-qPCR and plasmid construction.

Name	Forward primer (5′–3′)	Reverse primer (5′–3′)
CCNB1	GACCTGTGTCAGGCTTTCTCTG	GGTATTTTGGTCTGACTGCTTGC
CDC25A	TCTGGACAGCTCCTCTCGTCAT	ACTTCCAGGTGGAGACTCCTCT
CDC45C	TGGATGCTGTCCAAGGACCTGA	CAGGACACCAACATCAGTCACG
CDK1	GGAAACCAGGAAGCCTAGCATC	GGATGATTCAGTGCCATTTTGCC
MCM3	CGAGACCTAGAAAATGGCAGCC	GCAGTGCAAAGCACATACCGCA
MCM4	CTTGCTTCAGCCTTGGCTCCAA	GTCGCCACACAGCAAGATGTTG
MCM6	GACAACAGGAGAAGGGACCTCT	GGACGCTTTACCACTGGTGTAG
MCM7	GCCAAGTCTCAGCTCCTGTCAT	CCTCTAAGGTCAGTTCTCCACTC
FEN1	ACTAAGCGGCTGGTGAAGGTCA	GCAGCATAGACTTTGCCAGCCT
NEIL3	AGTGGTCTCCACCCAGCTGTTA	AGAGCAAGTCCTGCTTTACGGC
POLE	ACGCTGGAAGAGGTGTATGGCT	GGAACGGTTCTCAGAGATGAGC
POLE2	TGCGTCCGTTTTCCTAGCAGCA	GGGCAGACATAAAGAGGTAGGG
EXO1	TCGGATCTCCTAGCTTTTGGCTG	AGCTGTCTGCACATTCCTAGCC
RFC2	GTCGGGAATGAAGACACCGTGA	CAGAATGCTTGTGGTCTTGCCG
RFC3	CCTGAGACAGATTGGGAGGTGT	AGCTCATACAGCCTTCCACGAAC
RFC4	GGCAGCTTTAAGACGTACCATGG	TCTGACAGAGGCTTGAAGCGGA
Shctrl	CCGGCTCCGAACGTGTCACGCTC	AATTAAAAACTCCGAACGTGTCACGCTCGAGCGTGACACGTTCGGAG
	GAGCGTGACACGTTCGGAGTTTTT	AATTAAAAAGACCTGTGGACTCTCATATCTCGAGATATGAGAGTCCACAGGTC
shUSP32	CCGGGACCTGTGGACTCTCATATCTCGAGATATGAGAGTCCACAGGTCTTTTT	

### Western blotting

U-251 MG and U-87 MG cells were lysed using RIPA buffer (Vazyme). The protein concentrations of samples were measured using a bicinchoninic acid (BCA) protein quantification kit (Abcam, Shanghai, China). Samples (12 μg/lane) were loaded into a 12% SDS-PAGE gel for electrophoresis and then transferred onto a PVDF membrane (Roche, Basel, Switzerland). The membrane was incubated with primary antibody Anti-USP32 (1:1000, CAT#ab251903, Abcam) or Anti-GAPDH (1:2000, CAT#ab9485, Abcam) at 4 °C overnight, followed by incubation with secondary antibody HRP-conjugated Goat Anti-Rabbit IgG H&L (1:2000, CAT#ab6721, Abcam) at 28 °C for 30 min. The signals were visualized using the ECL detection system (Thermo Fisher Scientific, Waltham, MA, USA) and quantified by densitometry using Image J v1.48u.

### USP32 knockdown by transfection

U-251 MG and U-87 MG cells were seeded into 6-well plates at a density of 5 × 10^5^ per well and transfected with 200 pmol/well siRNA NC, siUSP32-2261, or siUSP32-386 using Lipofectamine RNAiMAX. The cells were harvested and used for further experiments after incubation for 6–48 h at 37 °C.

### CCK-8 assay for cell viability

U-251 MG and U-87 MG cells were tripsinized after a 24 h of transfection and then seeded into 96-well plates at a density of 5 × 10^3^ per well. At different time points (0, 24, 48, 72 h), 10 μL/well CCK-8 solution was added into cells and the cell viability was evaluated by detecting OD_450_.

### Colony formation assay for cell growth

U-251 MG and U-87 MG cells were tripsinized and then seeded into 6-well plates at a density of 1 × 10^3^ per well 6 h after transfection. The cells were continuously cultured at 37 °C for 7–10 days. The colonies were fixed with 4% methanal (Sangon Biotech, Shanghai, China) in phosphate buffer saline (PBS) at 28 °C for 15 min. After washing with PBS three times, the colonies were photographed using HUAWEI Mate 40 (Huawei, Shenzhen, China) and the number of colonies was counted using Image J v1.48u (NIH, Bethesda, MD, USA).

### Microscopic analysis of EdU incorporation for cell proliferation

U-251 MG and U-87 MG cells were tripsinized after a 24 h of transfection and then seeded into 96-well plates at a density of 2 × 10^4^ per well. Twelve hours later, the cells were labeled with 10 μM EdU solution (Beyotime, Shanghai, China) at 37 °C for 3 h. After fixation with 4% methanal in PBS at 28 °C for 20 min and subsequent permeation with 0.5% Triton X-100 (Sangon Biotech) in PBS at 28 °C for 10 min, the cells were incubated with 50 µL of 1 × Click Additive Solution (Beyotime) at 28 °C for 30 min. The nucleus was stained with 5 μg/mL DAPI (Yeasen). Finally, the fluorescent dots were observed and photographed using a fluorescence microscope (MOTIC, Hongkong, China) and then photographed. Image J v1.48u was used to count the number of cells.

### Flow cytometry-based cell cycle

U-251 MG and U-87 MG cells were collected after a 24 h of transfection and then fixed using 70% ethanol in PBS at − 20 °C for 6 h. The cells were then treated with 0.5% Triton X-100 and 10 μg/mL RNase (Sangon Biotech) at 28 °C for 25 min. Finally, the cells were stained with 20 μg/mL propidium iodide (PI, Vazyme) in the dark at 28 °C for 25 min and then placed into to the flow cytometer NovoCyte 1300 (ACEA, San Diego, CA, USA) for fluorescent detection within the PE-channel (Ex: 488 nm/Em: 578 nm).

### Transwell assay for cell migration and invasion

U-251 MG and U-87 MG cells were tripsinized after a 24 h of transfection and then suspended in FBS-free DMEM at a density of 3 × 10^5^/mL. In the transwell migration assay, 100 μL of suspended cells were placed into the upper chambers of transwell plates (NEST, Wuxi, Jiangsu, China). The lower chamber was supplied with 550 μL 10% FBS DMEM. In the Matrigel invasion assay, the membrane of the upper chamber was pre-coated with sixfold diluted Matrigel (Corning, Corning, NY, USA) before seeding cells. The migrated and invasive cells were fixed with 4% methanal in PBS and then stained with 1% crystal violet (Sangon Biotech) in PBS at 28 °C for 20 min, respectively. The stained cells were observed and photographed using a light microscope (MOTIC). The number of migrated/invasive cells were counted using Image J v1.48u in three random fields.

### Construction of stable U-87 MG cell lines with USP32 knockdown (shUSP32)

The lentiviral vector PLKO.1-TRC-Puro (Antihela, Xiamen, Fujian, China) was used to construct plasmid overexpressing short hairpin RNA targeting USP32 and shctrl plasmid. The primers for shUSP32 plasmid construction were designed based on the sequence of siUSP32-386 (Table 2). For lentiviral packaging, 293T cells (4 × 10^6^/well) were seeded into 6-well plates and transfected with 3 µg shUSP32 or shctrl plasmid, 2 µg psPAX2 (Antihela), and 1 µg pMD2.G (Antihela) using Lipofectamine RNAiMAX at 37 °C. After incubation for 48 h, the lentivirus was harvested and used to infect U-87 MG cells at a multiplicity of infection of 30 with the addition of 10 µg/mL polybrene (Thermo Fisher Scientific). Forty-eight hours after infection, U-87 MG cells were treated with 1.0 μg/mL puromycin (Yeasen) for 3 days, constructing shUSP32 and shctrl U-87 MG cells.

### Animal experiments

Six-week-old female BALB/c nude mice were obtained from Vitalriver (Beijing, China). The subcutaneous injection of shctrl or shUSP32 cells (4 × 10^6^) was performed into the right flank of six mice. The long diameter (a) and short diameter (b) of tumors were measured every 4 days. The tumor volume (V) was calculated using the formula V = ab^2^/2^26^. The mice were euthanized using isoflurane (RWD life science, Shenzhen, China) at day 48. Tumors were dissected off and photographed using HUAWEI Mate 40. The tumor was also weighed.

### RNA sequencing (RNA-Seq) and data analysis

Total RNA from shctrl or shUSP32 U-87 MG cells was used as input material for the RNA sample preparations. mRNA was purified from total RNA using poly-T oligo-attached magnetic beads. The RNA-Seq library was built by Novogene (Beijing, China). After cluster generation using TruSeq PE Cluster Kit v3-cBot-HS (Illumia, San Diego, CA, USA) on a cBot Cluster Generation System (Illumia), the library preparations were sequenced on a Novaseq platform (Illumina). RNA-Seq data analysis was performed according to the protocol of Novogene. In brief, reads were aligned to the human transcriptome and genome hg19 using T Hisat2 v2.0.5. Transcripts and genes were quantified using featureCounts v1.5.0-p3. Differential expression analysis was performed using DESeq2 R package v1.20.0. Genes with an adjusted *p* value < 0.05 were considered as differentially expressed. GO and KEGG pathway enrichment analyses^27–29^ of differentially expressed genes were performed using the clusterProfiler R package.

### Statistical analysis

All statistical analyses were performed using GraphPad Prism v8.2.1 (GraphPad Software, San Diego, CA, USA). The data are presented as mean ± standard deviation (SD) unless otherwise shown. ANOVA followed by Tukey’s post-hoc test was used for multiple comparisons among three groups. Unpaired Student's *t* test was performed to compare the difference between two groups. Survival curves were calculated using the Kaplan–Meier method, and the significance was determined by the log-rank test. Statistical significance was accepted at *p* < 0.05.

### Ethics approval

Animal experiments were conducted in accordance with the national guidelines for the humane treatment of animals and were approved by the Institutional Animal Care and Use Committee (IACUC) at Xiamen University. The study is reported in accordance with ARRIVE guidelines.

## Results

### High content screening

**Figure 1:**
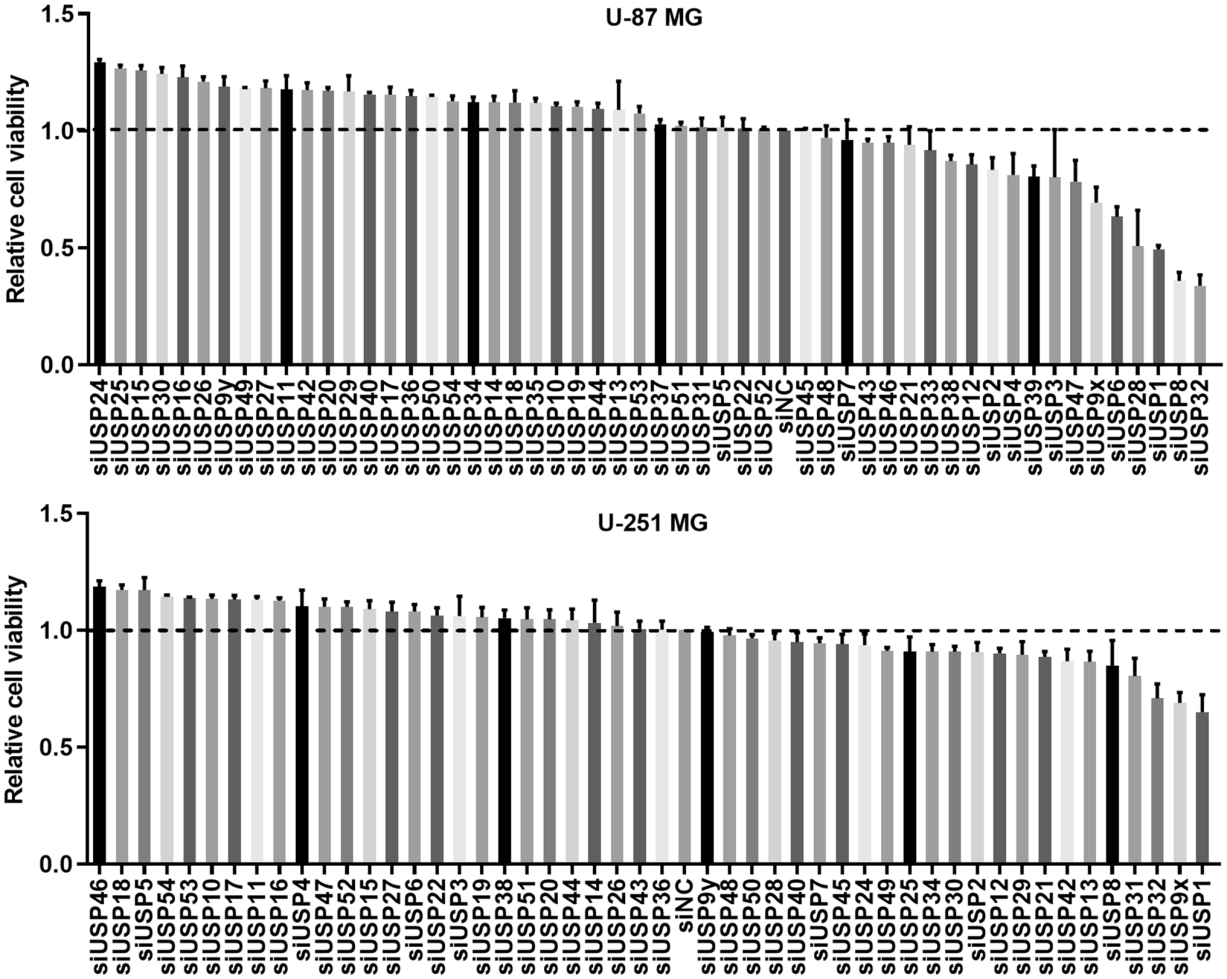
High content screening based on cell viability to screen USPs may function in glioblastoma. Data are represented as mean ± standard deviation (SD) of three biological replicates.

High content screening was performed to identify the USPs regulating GBM cell survival. As shown in Fig. 1, knockdown of USP1, USP8, or USP32 in U-87 MG cells inhibited cell viability by at least half. Knockdown of USP32, USP9X, or USP1 suppressed the viability of U-251 MG. Therefore, USP32 was chosen for further study.

### USP32 expression level linked to poor prognosis

**Figure 2:**
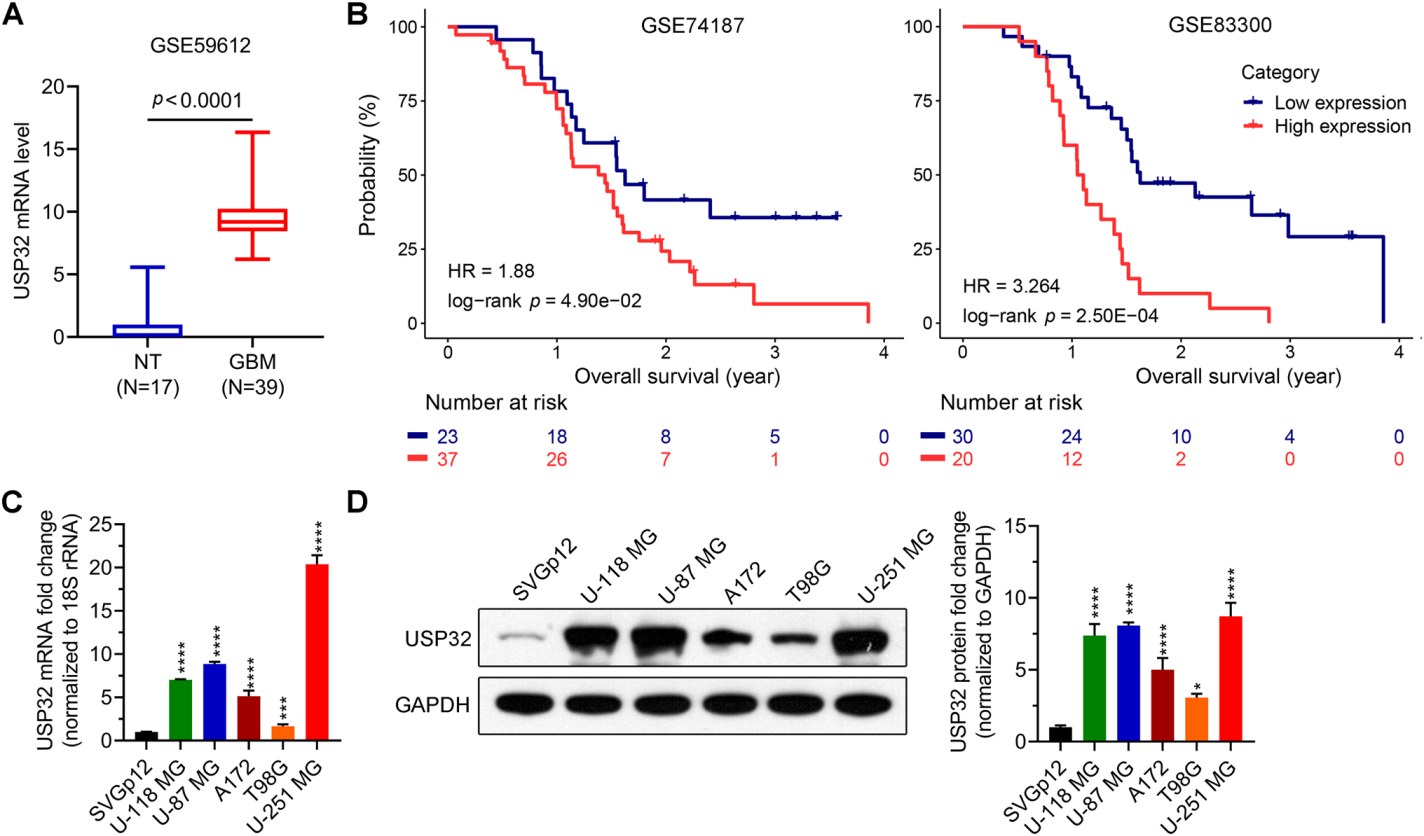
USP32 expression level in glioblastoma (GBM) tissues and cell lines and the association of USP32 expression level with prognosis. (**A**,**B**) USP32 is upregulated in GBM patients (**A**), which indicates poor prognosis (**B**). The USP32 mRNA expression data and survival information of GBM patients were downloaded from the Gene Expression Omnibus databases. (**C**,**D**) The USP32 mRNA and protein levels in GBM cells (U-118 MG, U-87 MG, A172, T198G, and U-251 MG) and normal brain cell SVG p12 were determined using RT-qPCR (**C**) and western blotting (**D**), respectively. Data are represented as mean ± SD of three technical replicates. Unpaired Student's *t* test for (**A**). Kaplan–Meier method and the significance was determined using the log-rank test for (**B**). One way ANOVA followed by Tukey’s post-hoc test for (**C**,**D**): vs SVG p12, *p < 0.05, ***p < 0.001, ****p < 0.0001.

First, we investigated the clinical significance of USP32 using GEO dataset. As can be seen in Fig. 2A, the USP32 expression level was higher in GBM tissues compared to normal tissues. Moreover, higher USP32 expression level indicated poorer prognosis (Fig. 2B). Next, we evaluated the USP32 expression level in normal brain cells and GBM cells. The results showed that the USP32 mRNA and protein levels in GBM cells (U-118 MG, U-87 MG, A172, T98G, and U-251 MG) were higher than those in the normal brain cell SVG p12 (Fig. 2C,D). Furthermore, U-87 MG and U-251 MG have the highest USP32 expression levels. Based on these findings, we chose to knock down USP32 in U-87 MG and U-251 MG cells to study the function of USP32 in GBM.

### Knockdown of USP32 inhibits cell growth

**Figure 3:**
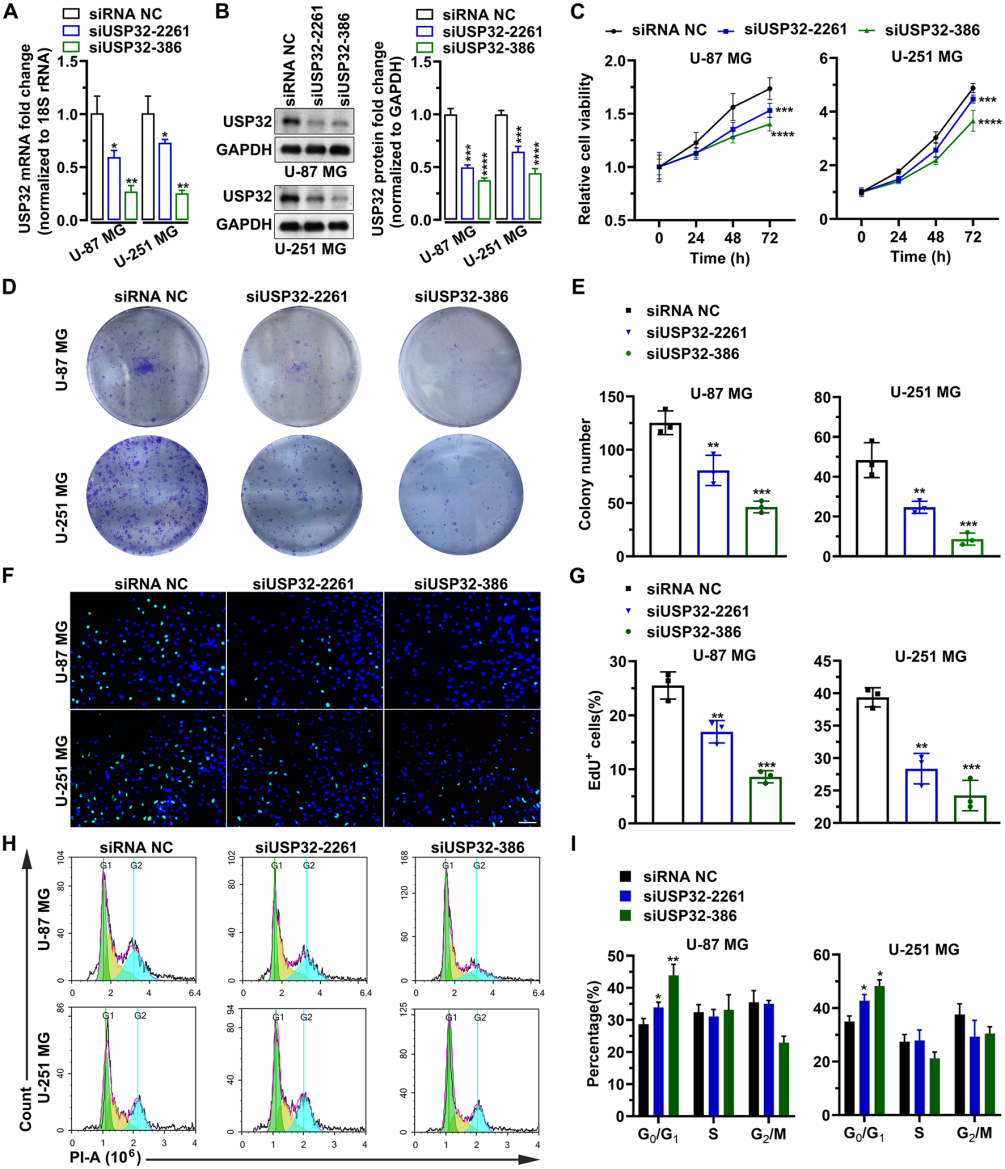
Knockdown of USP32 inhibits cell growth. (**A**) The mRNA level of USP32 was reduced by siUSP32-2261 and siUSP32-386. Data are represented as mean ± SD of three biological replicates. (**B**) Left panel: Representative images of three independent western bolting analyses showing the knockdown efficiency of siUSP32-2261 and siUSP32-386. Right panel: statistical quantification of left panel. (**C**) MTT assay determining the cell viability of U-87 MG and U-251 MG. Data are represented as mean ± SD of six biological replicates. (**D**) Representative images of three independent colony formation assays showing that USP32 knockdown inhibited cell proliferation. (**E**) Statistical quantification of (**D**). (**F**) USP32 knockdown reduced the number of EdU^+^ cells. Bar: 10 μm. (**G**) Statistical quantification of (**F**). Data are represented as mean ± SD of three technical replicates. (**H**) Representative images of three independent cell cycle assays by flow cytometry. (**I**) Histogram showing the percentage of each cell-cycle phase in (**H**). One way ANOVA followed by Tukey’s post-hoc test: vs siRNA NC, *p < 0.05, **p < 0.01, ***p < 0.001, ****p < 0.0001.

USP32 in U-87 MG and U-251 MG cells was knocked down using siRNAs. As shown in Fig. 3A,B, the mRNA and protein levels of USP32 were significantly reduced after transfection with siUSP32-2261 and siUSP32-386. Moreover, siUSP32-386 had higher knockdown efficiency than siUSP32-2261. Similarly, the CCK-8 assay indicated that USP32 knockdown significantly suppressed the viability of U-87 MG and U-251 MG cells (Fig. 3C). Cellular proliferation was analyzed using colony formation and EdU assays in U-87 MG and U-251 MG cells; Fig. 3D,E show that USP32 knockdown significantly reduced the number of colonies. In U-87 MG cells, the number of colonies decreased from 125 ± 11 to 80 ± 14 (*p* = 0.0058) for siUSP32-2261 and 46 ± 5 (*p* = 0.0003) for siUSP32-386. In U-251 MG cells, the number of colonies decreased from 48 ± 8 to 24 ± 3 (*p* = 0.0051) for siUSP32-2261 and 8 ± 3 (*p* = 0.0003) for siUSP32-386. The percentage of EdU^+^ cells was also significantly reduced by silencing USP32 (Fig. 3F). In U-87 MG cells, the percentage of EdU^+^ cells decreased from 25.5 ± 2.5% to 16.9 ± 2.0% (*p* = 0.0045) for siUSP32-2261 and 8.6 ± 1.1% (*p* = 0.0001) for siUSP32-386. In U-251 MG cells, the percentage of EdU^+^ cells decreased from 39.3 ± 1.4% to 28.3 ± 2.3% (*p* = 0.0016) for siUSP32-2261 and 24.2 ± 2.3% (*p* = 0.0003) for siUSP32-386 (Fig. 3G). Next, we investigated the effect of USP32 on cell cycle progression. The results show that USP32 knockdown promotes the arrest of cells in the G_0_/G_1_ phase (Fig. 3H,I). These findings suggested that silencing USP32 may inhibit cell growth due to cell-cycle arrest.

### Knockdown of USP32 inhibits cell metastasis

**Figure 4:**
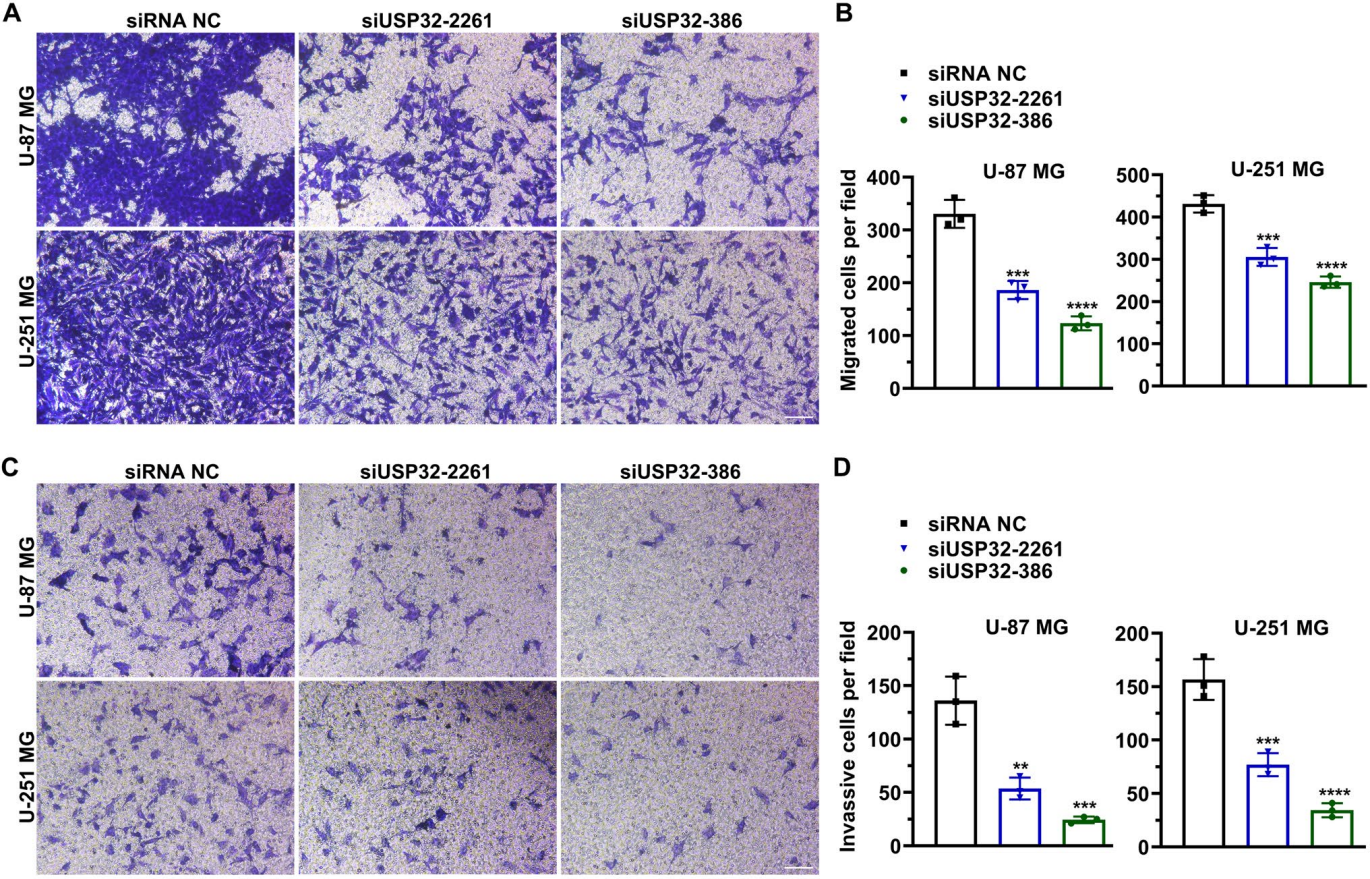
Knockdown of USP32 inhibits cell metastasis. (**A**) Transwell migration assay showing that silencing USP32 suppressed cell migration. Bar: 10 μm. Images were representatives of three independent experiments. (**B**) Statistical quantification of (**A**). (**C**) Matrigel invasion assay showing that USP32 deficiency suppressed cell invasion. Bar: 10 μm. Images were representatives of three independent experiments. (**D**) Statistical quantification of (**C**). One way ANOVA followed by Tukey’s post-hoc test: vs siRNA NC, **p < 0.01, ***p < 0.001, ****p < 0.0001.

Transwell migration and Matrigel invasion assays were performed to study the effect of USP32 on cell metastasis. Figure 4A shows that USP32 downregulation inhibited cell migration. In U-87 MG cells, the number of migrated cells was 330 ± 26, 186 ± 17, and 123 ± 13 for siRNA NC, siUSP32-2261 (*p* = 0.0003, vs siRNA NC), and siUSP-386 (*p* < 0.0001, vs siRNA NC), respectively. In U-251 MG cells, the number of migrated cells was 431 ± 20, 305 ± 21, and 246 ± 13 for siRNA NC, siUSP32-2261 (*p* = 0.0004, vs siRNA NC), and siUSP-386 (*p* < 0.0001, vs siRNA NC), respectively (Fig. 4B). This downregulation also inhibited cell invasion (Fig. 4C). In U-87 MG cells, the number of invasive cells decreased from 136 ± 22 to 53 ± 10 (*p* = 0.0010) for siUSP32-2261 and 24 ± 3 (*p* = 0.0002) for siUSP32-386. In U-251 MG cells, the number of invasive cells decreased from 136 ± 22 to 53 ± 10 (*p* = 0.0010) for siUSP32-2261 and 24 ± 3 (*p* = 0.0002) for siUSP32-386. In U-251 MG cells, the number of invasive cells were reduced from 156 ± 19 to 77 ± 10 (*p* = 0.0008) for siUSP32-2261 and 34 ± 6 (*p* < 0.0001) for siUSP32-386 (Fig. 4D). Taken together, these data indicate that USP32 knockdown inhibited cell metastasis.

### Knockdown of USP32 inhibits tumor growth in vivo

**Figure 5:**
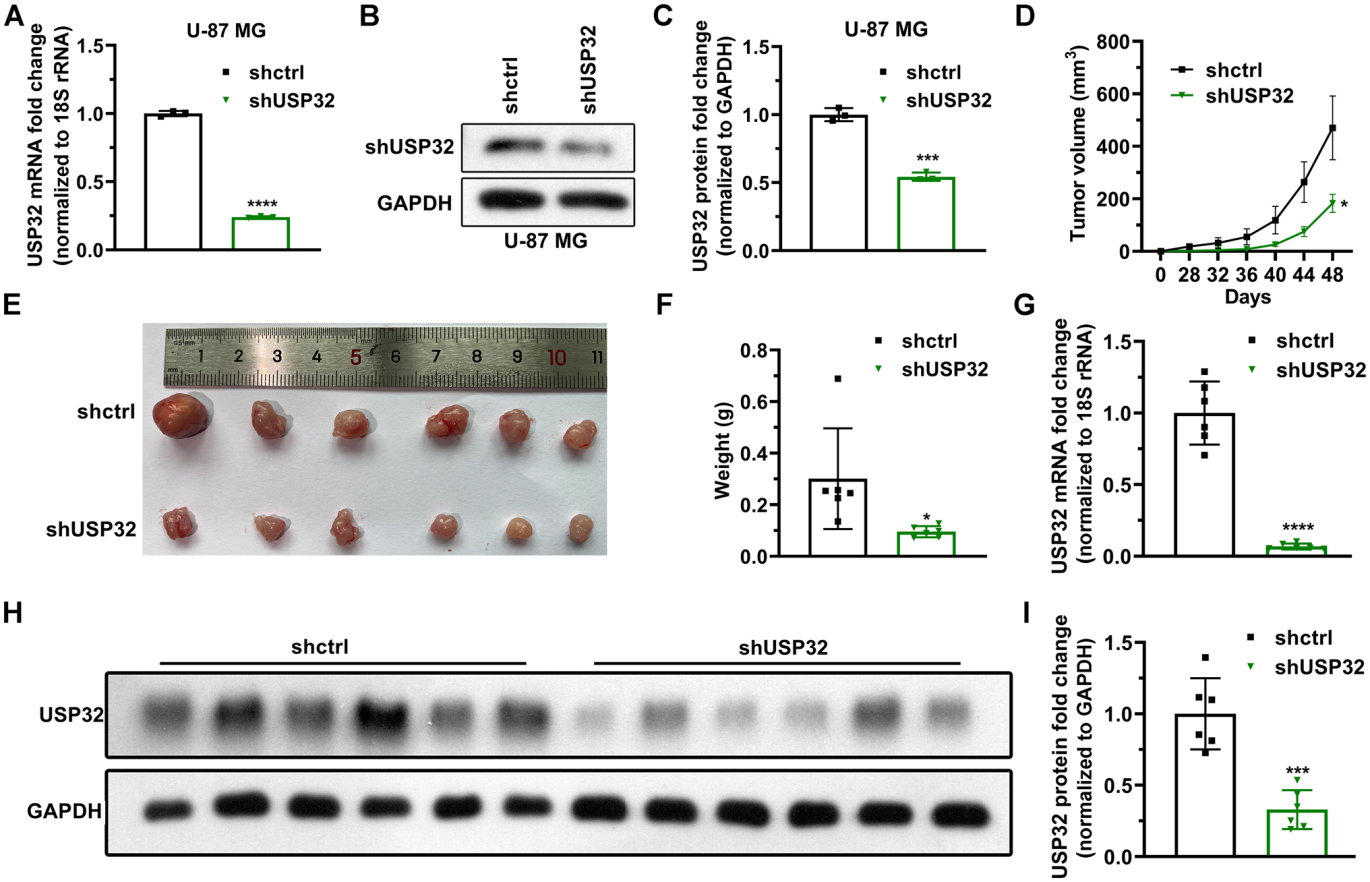
Knockdown of USP32 suppresses tumor growth in vivo. (**A**,**C**) Stably USP32-knockdown U-87 MG cell lines were successfully constructed, determined using RT-qPCR (**A**) and western blotting (**B**,**C**). Data are represented as mean ± SD of three technical replicates. (**D**) Tumor volume was tracked every 4 days by calculating using the formula: volume = long diameter × short diameter^2^/2. Data are represented as mean ± SD of six mice. (**E**) The image of tumors. The mice were euthanized using isoflurane at day 48 and tumors were dissected off. (**F**) USP32 knockdown reduced the weight of tumors. Data are represented as mean ± SD of six mice. (**G**) RT-qPCR analysis degerming the mRNA level of USP32 in tumor tissues. Data are represented as mean ± SD of six mice. (**H**) Western blotting. (**I**) Statistical quantification of (**H**). Unpaired Student's *t* test: vs shctrl, *p < 0.05, ***p < 0.001, ****p < 0.0001.

Next, stably USP32-knockdown U-87 MG cells were constructed to validate the function of USP32 in vivo. Figure 5A–C show that the stable cell lines were successfully constructed. The constructed cells were then subcutaneously injected into nude mice. As can be seen in Fig. 5D, the tumor in group shUSP32 grew more slowly compared to group shctrl. The dissected tumors are shown in Fig. 5E, being the weight lighter when group shUSP32 was compared to shctrl (Fig. 5F). Moreover, the mRNA and protein levels of USP32 were indeed lower in group shUSP32 than those in group shctrl (Fig. 5G–I). These data demonstrate that USP32 knockdown inhibits tumor growth in vivo.

### Analysis of differentially expressive genes (DEGs)

**Figure 6:**
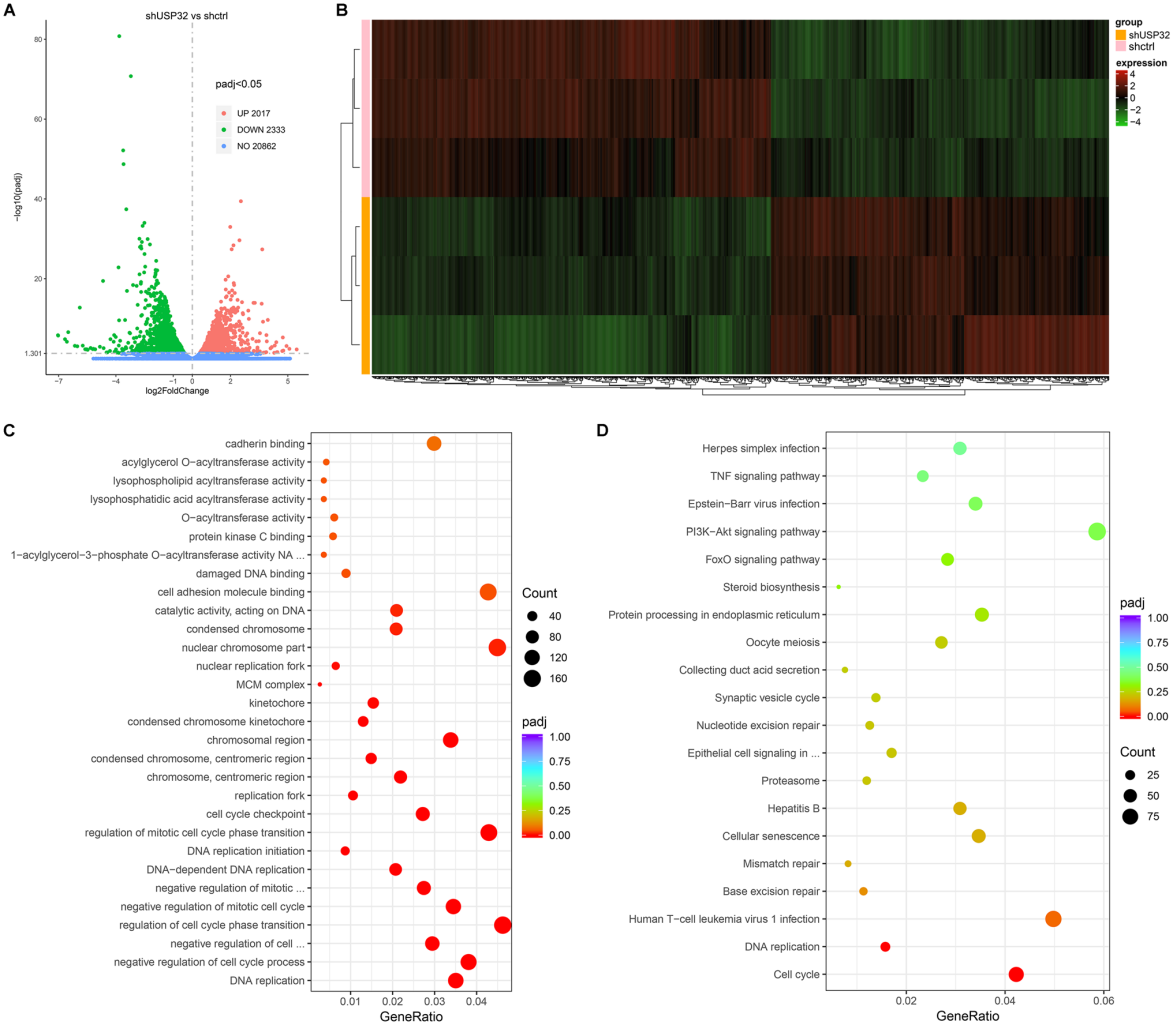
Gene Ontology (GO) and Kyoto Encyclopedia of Genes and Genomic (KEGG) pathway enrichment analyses using differentially expressive genes (DEGs) screened out by transcriptional sequencing. (**A**) Volcano plot based on the results of RNA sequencing for transcriptomes from shctrl and shUSP32 U87-MG cells. (**B**) The heat map showing the distinguishable mRNA expression patterns between the shUSP32 and shctrl samples. (**C**) GO enrichment analysis showing the considerable molecular functions, cellular components, and biological processes. (**D**) KEGG pathway enrichment analysis showing the considerable pathways.

Transcriptional sequencing was performed to find the differentially expressive genes between shctrl and shUSP32 U87-MG cells. The volcano plot indicates that 2017 genes were significantly upregulated and 2333 genes were significantly downregulated after USP32 knockdown (Fig. 6A). The heat map was used to show the distinguishable mRNA expression patterns between shUSP32 and shctrl samples (Fig. 6B).

### GO and KEGG pathway enrichment analyses

**Figure 7:**
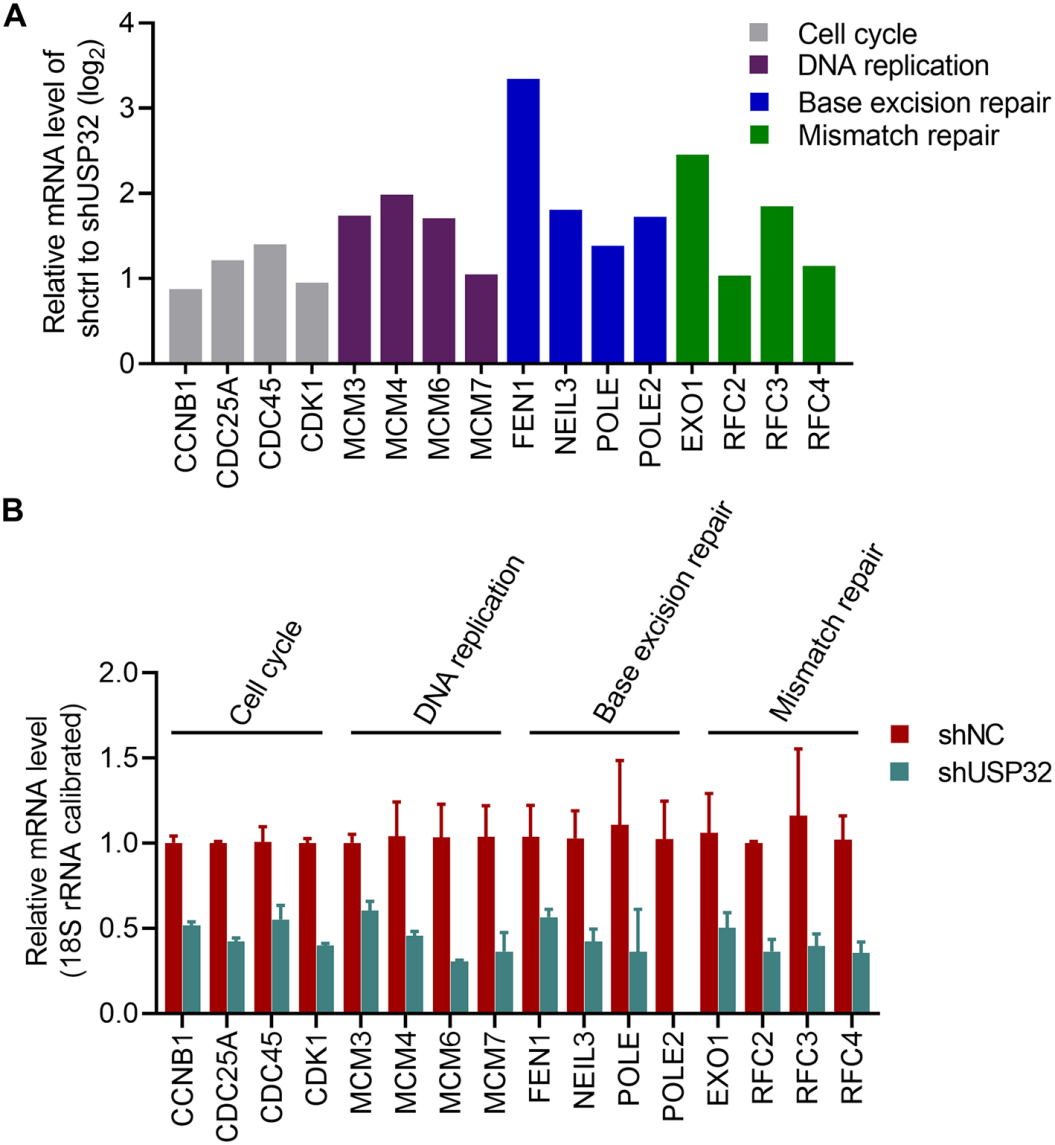
USP32 is involved in cell cycle, DNA replication, base excision repair, and mismatch repair processes/pathways. (**A**) The expression profile of several DEGs. (**B**) RT-qPCR analysis confirming the regulatory effect of USP32 on cell cycle, DNA replication, base excision repair, and mismatch repair processes/pathways. Data are represented as mean ± SD of three technical replicates.

All upregulated and downregulated genes were used for GO and KEGG enrichment analyses. In the GO enrichment analysis, 10 molecular functions such as cadherin binding and catalytic activity acting on DNA, 11 cellular components such as condensed chromosome and cell cycle checkpoint, and 9 biological processes such as mitotic cell cycle phase initiation and DNA replication were significantly modulated (Fig. 6C). KEGG pathway enrichment analysis discovers 20 pathways significantly linked to USP32 expression, including cell cycle and DNA replication pathways (Fig. 6D). The enriched DEGs in GO and KEGG pathway enrichment analyses are listed in Supplementary Table S1 and Supplementary Table S2, respectively. The expression profile of several DEGs associated with cell cycle, DNA replication, base excision repair, and mismatch repair are shown in Fig. 7A. RT-qPCR analysis confirmed that USP32 knockdown reduced the expression of these genes (Fig. 7B).

## Discussion

GBM accounts for 16% of primary brain tumors with an incidence rate of thirty-two per million population^1,2^. GBM often causes poor prognosis and the median-survival time of patients is less than 2 years^30,31^. It is urgent to find novel targets for brain-penetrating targeted therapies. Recently, more and more USPs were reported to play important roles in GBM progression^7,21^. USP1, USP8, USP9x, and USP28 were identified as oncogenes in GBM^32–35^. USPs with antitumor activity such as USP11 and USP286 promote cell viability after being silenced^36,37^, which is consistent with the results in Fig. 1. In this study, USP32 knockdown inhibited cell growth and metastasis in vitro, and suppressed tumor growth in vivo, which suggests that USP32 acts as an oncogene in GBM and may serve as a potential target for GBM treatment.

EdU^+^ cells indicate the cells in DNA replication. DNA replication, occurring in the S phase of interphase during cell cycle, is an important step for cell proliferation and division^38^. Results showed that the percentages of EdU^+^ cells and G_0_/G_1_-phase cells were reduced and increased after USP32 knockdown, respectively. Moreover, GO and KEGG pathway analyses revealed that this enzyme is involved in DNA replication and cell cycle processes or pathways. This suggests that USP32 may promote cells passing through the G_0_/G_1_ phase and initiate the DNA replication, promoting the proliferation of cancer cells, which is consistent with the study of Hu et al.^23^.

Base excision repair is an essential genome-maintenance pathway by which cells repair damaged DNA bases that arise at a high level during DNA replication. Failure to remove the damaged DNA bases causes increasing levels of mutation and chromosomal instability, finally resulting in carcinogenesis^39^. DNA mismatch repair is a rescue system that conserves the DNA sequences by removing the erroneously mismatched, inserted, and deleted bases during DNA duplication and recombination. Defects in DNA mismatch repair are also associated with carcinogenesis^40^. Moreover, DUBs are often involved in base excision repair and mismatch repair processes^17,18^. Therefore, USP32 effect on the expression of molecules functioning in these processes was validated using RT-qPCR, although the adjusted *p*-values for base excision repair and mismatch repair pathways in KEGG pathway analysis were greater than 0.05. The results indicate that USP32 regulates the expressions of MCM3, MCM4, MCM6, MCM7, FEN1, NEIL3, and POLE, suggesting that USP32 plays an important role in base excision repair and mismatch repair.

There are few reports about elements of the ubiquitin–proteasome system involved in GBM metastasis. USP18 was reported to promote epithelial-mesenchymal transition in GBM cells by deubiquitinating and stabilizing Twist1^41^. UBE2T, a ubiquitin-conjugating enzyme, stabilizes GRP78 to promote the metastasis of GBM cells^42^. Our study demonstrates that USP32 facilitates the migration and invasion of GBM cells, which supports that the ubiquitin–proteasome system plays an important role in the GBM metastasis.

Further experiments are needed to confirm the mechanism of action by which USP32 upregulates the expression of several genes (Fig. 7B). Further, the effect of USP32 modulation on cell function in normal glial cells and a broader panel of GBM cells will be evaluated. In addition, a collection of clinical samples to analyze the expression of this enzyme in GBM and normal tissue will also be included.

In conclusion, our study demonstrates that USP32 acts as an oncogene in GBM through regulating cell cycle, DNA replication, base excision repair, and mismatch repair. USP32 could be a potential target for GBM treatment.

## Supplementary Information

Supplementary Figures.

Supplementary Table S1.

Supplementary Table S2.

## Data Availability

The data in this study will be made available from the corresponding author on reasonable request.
