# Supplementary figures and images for: Identification of ubiquitin-specific protease 32 as an oncogene in glioblastoma and the underlying mechanisms
Source: Sci Rep. 2022 Apr 19;12:6445. doi: 10.1038/s41598-022-09497-y (PMC9018837; doi:10.1038/s41598-022-09497-y)

Figure 2D

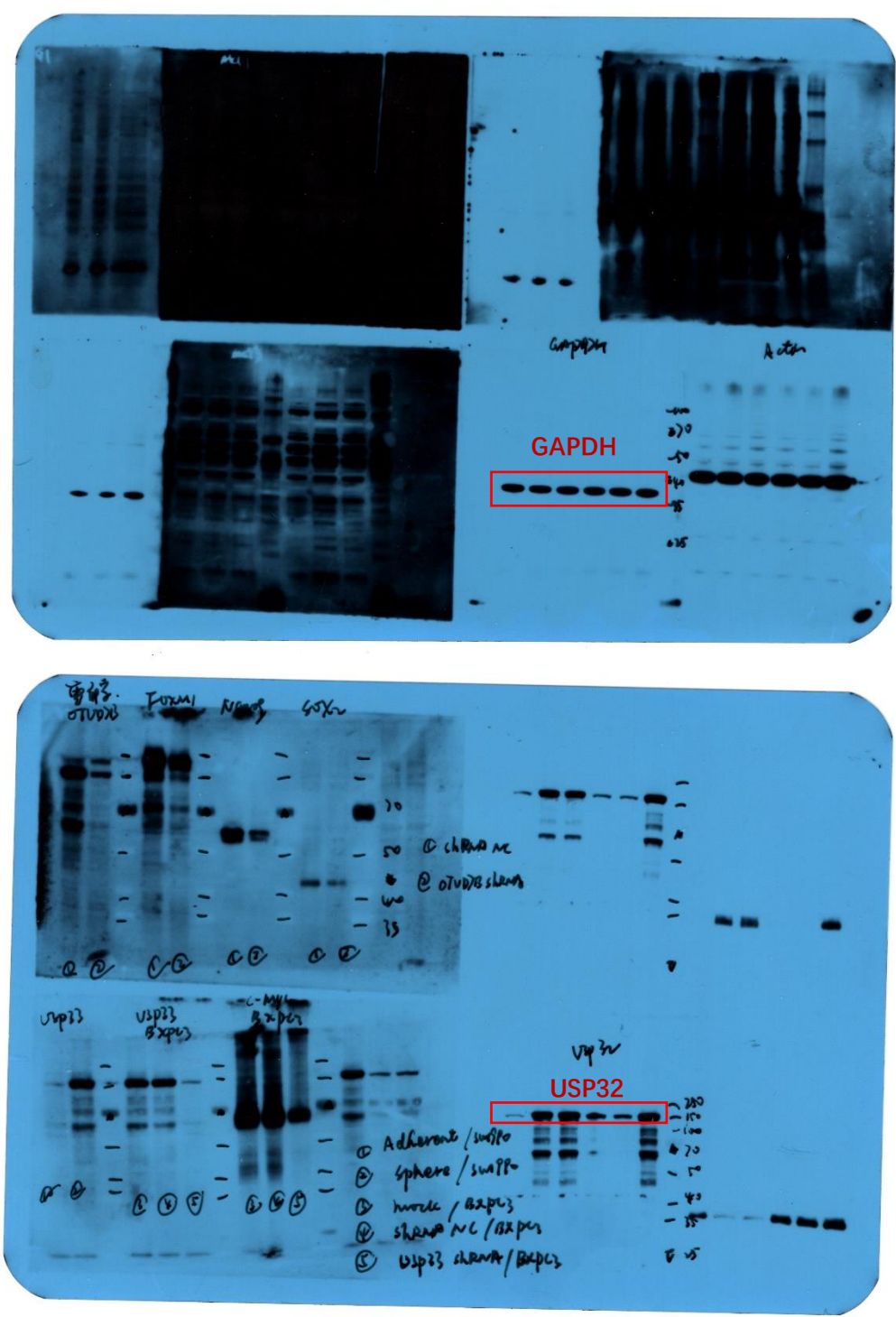

Figure 3B

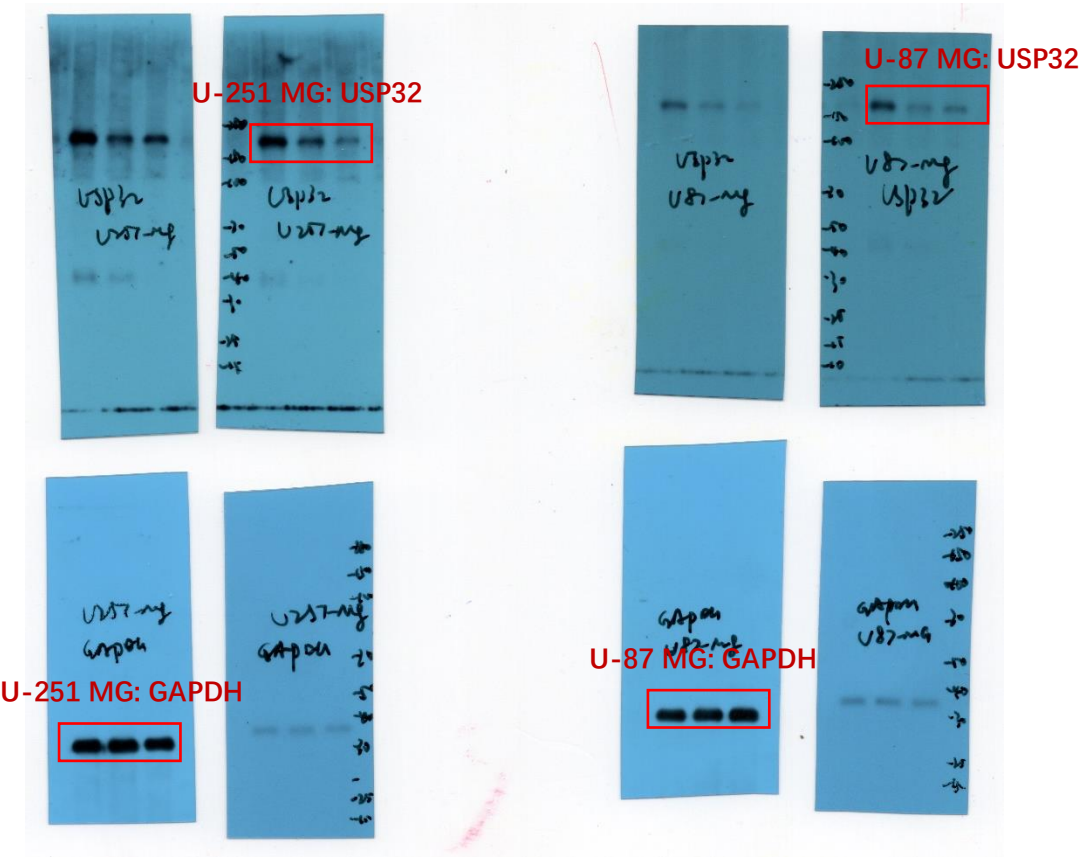

Figure 5H

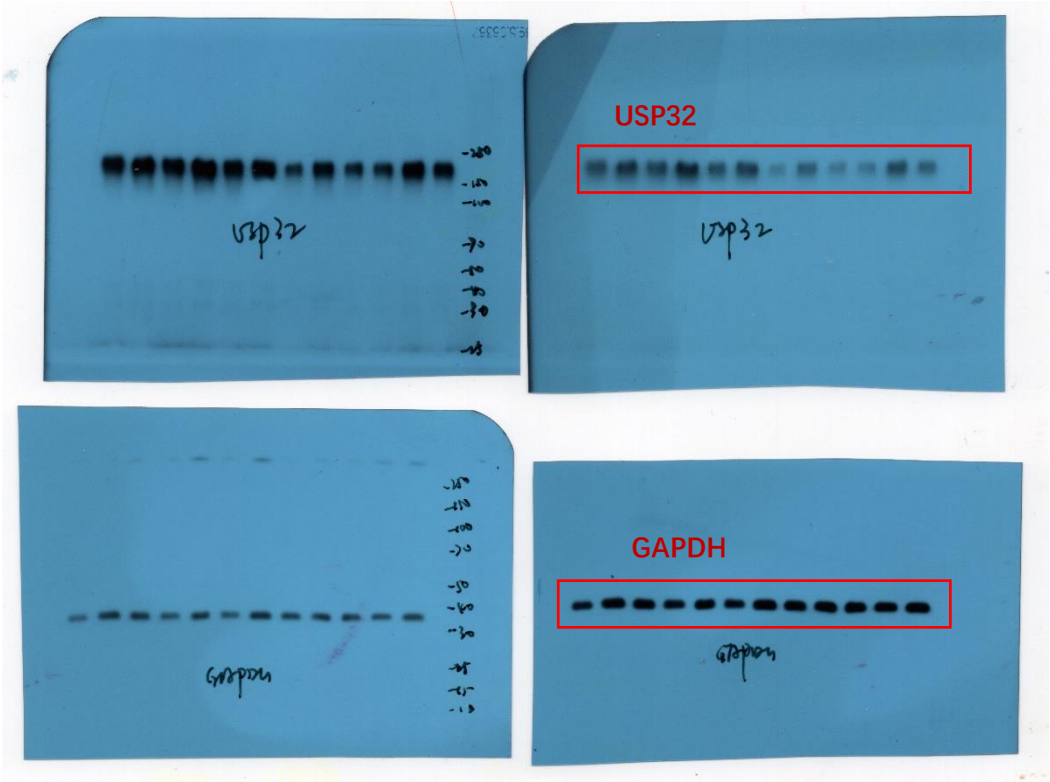

Supplement: Supplementary file 1 — Supplementary Figures. [file 41598_2022_9497_MOESM1_ESM.pdf]
