# Supplementary material for: Identification of ubiquitin-specific protease 32 as an oncogene in glioblastoma and the underlying mechanisms
Source: Sci Rep. 2022 Apr 19;12:6445. doi: 10.1038/s41598-022-09497-y (PMC9018837; doi:10.1038/s41598-022-09497-y)
Supplement: Supplementary file 2 — Supplementary Table S1. [file 41598_2022_9497_MOESM2_ESM.pdf]

Table S1. Gene Ontology (GO) enrichment analysis

Functional category	Significant DEGs
DNA replication (GO:0006260)	RRM2,MCM4,EREG,FEN1,MCM3,GINS2,CDC6,ORC6,RRM1,ORC1,GMNN,CDC45,DTL,MCM10,E2F8,GINS1,PCLAF,TIMELESS,PCNA,MCM6,CDC25A,RFC3,EXO1,PDGFA,CLSPN,NFIX,CDC7,NFIC,GINS4,CHAF1B,MCM7,BCL6,ESCO2,POLA2,GINS3, RFC4,MCM2,TICRR,HMGA1,POLE,TGFB1,DSCC1,CDK1,PTMS,RFWD3,CDK2,CHAF1A,POLD4,NASP,SLBP,MCM8,MCM5, DONSON,MCMBP,DNA2,BRCA1,RFC2,CCNE2,BARD1,NUCKS1,SMC1A,KCTD13,TOP1MT,PPP2CA,MUS81,RFC5,BRIP1,ORC5, TOPBP1,WDHD1,WDR18,UCN,FGFR1,MMS22L,LIG1,PARP1,POLE3,RBBP4,ID3,CACYBP,NBN,CDC34,POLL,RPA1,CTC1, ORC3,KAT5,POLE2,RMI1,CST3,S100A11,WIZ,DBF4,POLA1,BLM,RPA2,RMI2,CDK9,CALR,GLI1,TONSL,FAM111A, CDC25C,TIPIN,RBBP8,RAD1,PARP3,PRIM2,TOP1,POLQ,POLD3,HELB,HUS1,PURA,ING4,PNKP,CHEK1,POLD1, USP37, POLG,IGF1R,DACH1,ATRIP,INSR,RECQL4
negative regulation of cell cycle process (GO:0010948)	RRM2,LIF,MLXIPL,DTL,E2F8,PCNA,FBXO5,TRIP13,GADD45A,CLSPN,CDC7,KLF4,PSMC2,TFDP1,BRSK1,PCGF2,BCL6, DUSP1,FANCI,TICRR,AURKA,CCNB1,PSMD14,EZH2,CDK1,RFWD3,PLK3,PSMD12,PLK2,CDK2,NOP53,PSMD2, NDC80, DACT1,E2F1,TNKS1BP1,MAD2L1,CASP2,RBL1,DONSON,FOXO4,PSMB10,BRCA1,PSMD1,SMC1A,PSMA4,MSH2,PSMD11, GEN1,ZNF207,ZNF385A,GTSE1,CNOT9,MUS81,MTBP,CDKN1A,TOPBP1,UBA52,PIDD1,PSMD6,SKP1,RB1,CNOT6,PML, CTDSP1,RBBP4,BUB1B,PSMA1,MIIP,PSME4,BUB3,RINT1,NBN,HMGA2,PSMD7,DAB2IP,TP53,ARID3A,CTDSPL,RBX1, PSMA3,PSMD5,PRKDC,CARM1,AURKAIP1,PCBP4,PSMB1,BLM,ZFP36L1,RPA2,TP63,CDK9,GPNMB,CALR,TRI

	AP1,H2AFY,CDC25C,BUB1,RBBP8,TFAP4,CHMP2A,RAD1,MDM1,FBXO43,PLK1,CDC14B,GPER1,BMI1,PSME3,ABRAXAS1,BAX,VPS4A,HSP90AB1,PPP2R5B,PSMC5,EPC1,HUS1,PSMA2,CTDSP2,TTK,NEK2,CNOT7,PSMD10,CHEK1,CNOT10,PTTG1,FZR1,CNOT8,PHC1,HMGN5,CDKN2D,TMEM67,PRMT1,PSMA5,PSMC6,NAE1
negative regulation of cell cycle phase transition (GO:1901988)	DTL,E2F8,PCNA,TRIP13,GADD45A,CLSPN,KLF4,PSMC2,TFDP1,BRSK1,DUSP1,FANCI,TICRR,AURKA,CCNB1,PSMD14,EZH2,CDK1,RFWD3,PLK3,PSMD12,PLK2,CDK2,NOP53,PSMD2,NDC80,DACT1,E2F1,TNKS1BP1,MAD2L1,CASP2,RBL1,DONSON,FOXO4,PSMB10,BRCA1,PSMD1,PSMA4,PSMD11,GEN1,ZNF207,ZNF385A,GTSE1,CNOT9,MUS81,CDKN1A,TOPBP1,UBA52,PIDD1,PSMD6,SKP1,RB1,CNOT6,PML,CTDSP1,BUB1B,PSMA1,MIIP,PSME4,BUB3,RINT1,NBN,HMGA2,PSMD7,TP53,ARID3A,CTDSPL,RBX1,PSMA3,PSMD5,PRKDC,CARM1,PCBP4,PSMB1,BLM,ZFP36L1,RPA2,TP63,GPNMB,TRIAP1,H2AFY,CDC25C,BUB1,PLK1,CDC14B,PSME3,ABRAXAS1,BAX,VPS4A,PSMC5,HUS1,PSMA2,CTDSP2,TTK,CNOT7,PSMD10, CHEK1,CNOT10,FZR1,CNOT8,CDKN2D,PRMT1,PSMA5,PSMC6,NAE1

regulation of cell cycle phase transition<br>(GO:1901987)	ID2,CDC6,TCIM,PKD1,CDC45,DTL,E2F8,PCNA,CDC25A,TRIP13,CEP78,GADD45A,CLSPN,CDC7,KLF4,PSMC2,TFDP1,<br>PRKAR2B,BRSK1,DUSP1,HSP90AA1,FANCI,TICRR,TGFB1,AURKA,CCNB1,PSMD14,EZH2,CDK1,FAM83D,RFWD3,PLK3,<br>PSMD12,PLK2,CDK2,NOP53,PSMD2,NDC80,DACT1,PKIA,TPX2,CDC23,PLK4,E2F1,TNKS1BP1,MAD2L1,CASP2,RBL1,<br>DONSON,FOXO4,CSNK1E,PSMB10,BRCA1,PSMD1,PSMA4,SMARCD3,CDCA5,PSMD11,ATAD5,GEN1,CEP76,ZNF207,ZNF385A,GTSE1,CNOT9,MUS81,MTBP,CDKN1A,TOPBP1,KNTC1,UBA52,PIDD1,ERCC2,PSMD6,SKP1,RB1,BID,CNOT6,UBE2C,PML,<br>CTDSP1,BUB1B,PSMA1,MIIP,PSME4,BUB3,YWHAG,RINT1,XPC,NBN,ATP2B4,TUBA4A,HMGA2,CTC1,PSMD7,DLGAP5,TP53,ARID3A,CTDSPL,RBX1,PSMA3,PSMD5,PRKDC,CARM1,TMOD3,RAD51C,PCBP4,PSMB1,TUBA1A,HMMR,BLM,NEDD1,ZFP36L1,ECD,RPA2,TP63,GPNMB,TRIAP1,GLI1,RDX,MAPRE1,H2AFY,CDC25C,BUB1,PAXIP1,TFAP4,UBE2E2,ANAPC5,HAUS8,<br>DDRGK1,CENPE,HECW2,PLK1,CDC14B,FGFR1OP,PSME3,ABRAXAS1,BAX,VPS4A,PSMC5,HUS1,PSMA2,PPP1R9B,STXBP4,<br>CEP250,CTDSP2,TTK,NEK2,CNOT7,PSMD10,CHEK1,HAUS1,CNOT10,CDK6,ANXA1,CEP192,FZR1,CNOT8,DYNC1I2, CDKN2D,PRMT1,PSMA5,PSMC6,DYNLL1,NAE1

negative regulation of mitotic cell cycle (GO:0045930)	E2F8,PCNA,ZWILCH,TRIP13,GADD45A,BCL2L1,CLSPN,FOXC1,KLF4,PSMC2,TFDP1,BRSK1,BCL6,DUSP1,FANCI,TICRR, TGFB1,AURKA,CCNB1,PSMD14,EZH2,CDK1,RFWD3,PLK3,PSMD12,BTG1,PLK2,CDK2,NOP53,PSMD2,NDC80,DACT1,E2F1, TNKS1BP1,MAD2L1,CASP2,RBL1,DONSON,FOXO4,PSMB10,BRCA1,PSMD1,PSMA4,MSH2,PSMD11,GEN1,ZNF207,ZNF385A, GTSE1,CNOT9,MUS81,MTBP,CDKN1A,TOPBP1,KNTC1,UBA52,PIDD1,PSMD6,SKP1,RB1,CNOT6,PML,CTDSP1,ZWINT, BUB1B,PSMA1,MIIP,PSME4,BUB3,NLE1,RINT1,XPC,NBN,HMGA2,PSMD7,TP53,ARID3A,CTDSPL,RBX1,PSMA3,PSMD5, PRKDC,CARM1,ABL1,AURKAIP1,PCBP4,PSMB1,BLM,CTNNB1,ZFP36L1,BTG3,RPA2,TP63,GPNMB,TRIAP1,CDC25C,TIPIN,BUB1,ATF2,PLK1,CDC14B,PSME3,BAX,VPS4A,PSMC5,HUS1,PSMA2,CTDSP2,TTK,PNPT1,CNOT7,PSMD10,PRCC,CHEK1, CNOT10,PTTG1,CNOT8,PTPN3,NABP1,PRMT1,PSMA5,PSMC6,NAE1
negative regulation of mitotic cell cycle phase transition (GO:1901991)	E2F8,PCNA,TRIP13,GADD45A,CLSPN,KLF4,PSMC2,TFDP1,BRSK1,DUSP1,FANCI,TICRR,AURKA,CCNB1,PSMD14,EZH2, CDK1,RFWD3,PLK3,PSMD12,PLK2,CDK2,NOP53,PSMD2,NDC80,DACT1,E2F1,TNKS1BP1,MAD2L1,CASP2,RBL1,DONSON, FOXO4,PSMB10,BRCA1,PSMD1,PSMA4,PSMD11,GEN1,ZNF207,ZNF385A,GTSE1,CNOT9,MUS81,CDKN1A,TOPBP1,UBA52, PIDD1,PSMD6,SKP1,RB1,CNOT6,PML,CTDSP1,BUB1B,PSMA1,MIIP,PSME4,BUB3,RINT1,NBN,HMGA2,PSMD7,TP53, ARID3A,CTDSPL,RBX1,PSMA3,PSMD5,PRKDC,CARM1,PCBP4,PSMB1,BLM,ZFP36L1,RPA2,TP63,GPNMB,TRIAP1,CDC25C, BUB1,PLK1,PSME3,BAX,VPS4A,PSMC5,HUS1,PSMA2,CTDSP2,TTK,CNOT7,PSMD10,CNOT10,CNOT8,PRMT1,PSMA5,PSMC6,NAE1

DNA-dependent DNA replication (GO:0006261)	MCM4,FEN1,MCM3,GINS2,CDC6,ORC6,ORC1,GMNN,CDC45,MCM10,E2F8,GINS1,TIMELESS,PCNA,MCM6,RFC3,CDC7, GINS4,MCM7,BCL6,POLA2,GINS3,RFC4,MCM2,TICRR,HMGA1,POLE,DSCC1,RFWD3,CDK2,POLD4,SLBP,MCM8,MCM5, DONSON,MCMBP,DNA2,RFC2,CCNE2,NUCKS1,SMC1A,MUS81,RFC5,ORC5,TOPBP1,WDHD1,WDR18,FGFR1,MMS22L,LIG1, PARP1,POLE3,NBN,CDC34,RPA1,ORC3,POLE2,WIZ,POLA1,BLM,RPA2,CDK9,TONSL,TIPIN,PARP3,PRIM2,POLQ,POLD3, HELB,PURA,PNKP,POLD1,POLG,DACH1
DNA replication initiation(GO:0006270)	MCM4,MCM3,GINS2,CDC6,ORC6,ORC1,CDC45,MCM10,MCM6,CDC7,GINS4,MCM7,POLA2,MCM2,TICRR,POLE,CDK2,MCM8, MCM5,CCNE2,ORC5,TOPBP1,WDR18,POLE3,NBN,CDC34,ORC3,POLE2,POLA1,PRIM2,PURA
regulation of mitotic cell cycle phase transition (GO:1901990)	ID2,CDC6,PKD1,CDC45,DTL,E2F8,PCNA,TRIP13,CEP78,GADD45A,CLSPN,CDC7,KLF4,PSMC2,TFDP1,PRKAR2B,BRSK1, DUSP1,HSP90AA1,FANCI,TICRR,TGFB1,AURKA,CCNB1,PSMD14,EZH2,CDK1,RFWD3,PLK3,PSMD12,PLK2,CDK2,NOP53, PSMD2,NDC80,DACT1,PKIA,TPX2,CDC23,PLK4,E2F1,TNKS1BP1,MAD2L1,CASP2,RBL1,DONSON,FOXO4,CSNK1E,PSMB10,BRCA1,PSMD1,PSMA4,SMARCD3,CDCA5,PSMD11,ATAD5,GEN1,CEP76,ZNF207,ZNF385A,GTSE1,CNOT9,MUS81,MTBP, CDKN1A,TOPBP1,KNTC1,UBA52,PIDD1,ERCC2,PSMD6,SKP1,RB1,BID,CNOT6,UBE2C,PML,CTDSP1,BUB1B,PSMA1,MIIP, PSME4,BUB3,YWHAG,RINT1,XPC,NBN,TUBA4A,HMGA2,CTC1,PSMD7,DLGAP5,TP53,ARID3A,CTDSPL,RBX1,PSMA3,PSMD5,PRKDC,CARM1,TMOD3,RAD51C,PCBP4,PSMB1,TUBA1A,HMMR,BLM,NEDD1,ZFP36L1,ECD,RPA2,TP63,GPNMB,TRIAP1,RDX,MAPRE1,CDC25C,BUB1,TFAP4,UBE2E2,ANAPC5,HAUS8,CENPE,HECW2,PLK1,CDC14B,FGFR1OP,PSME3,BAX,VPS4A, PSMC5,HUS1,PSMA2,PPP1R9B,CEP250,CTDSP2,TTK,NEK2,CNOT7,PSMD10,HAUS1,CNOT10,CDK6,ANXA1,CEP192,CNOT8,DYNC1I2,CDKN2D,PRMT1,PSMA5,PSMC6,DYNLL1,NAE1

cell cycle checkpoint (GO:0000075)	WDR76,CDC6,CDC45,DTL,E2F8,TIMELESS,PCNA,ZWILCH,TRIP13,GADD45A,BCL2L1,CLSPN,TFDP1,BRSK1,DUSP1, FANCI,TICRR,TGFB1,AURKA,CCNB1,CDK1,RFWD3,PLK3,PLK2,CDK2,NOP53,NDC80,E2F1,TNKS1BP1,MAD2L1,CASP2, DONSON,FOXO4,INTS7,DNA2,BRCA1,MSH2,GEN1,ZNF207,ZNF385A,GTSE1,CNOT9,MUS81,CDKN1A,BRIP1,TOPBP1, KNTC1,UBA52,PIDD1,RB1,CNOT6,SPDL1,PML,ZWINT,BUB1B,BUB3,RINT1,XPC,NBN,HMGA2,TP53,ARID3A,H2AFX, PRKDC,CARM1,PCBP4,BLM,RPA2,TP63,TRIAP1,CDC25C,TIPIN,BUB1,RBBP8,ATF2,RAD1,PLK1,CDC14B,ABRAXAS1, BAX,VPS4A,HUS1,FBXO4,TTK,CNOT7,PRCC,CHEK1,CNOT10,FZR1,CNOT8,DCLRE1B,ATRIP,TIPRL,NABP1,DDX39B, PRMT1,NAE1
mitotic cell cycle checkpoint (GO:0007093)	E2F8,PCNA,ZWILCH,TRIP13,GADD45A,BCL2L1,CLSPN,TFDP1,BRSK1,DUSP1,FANCI,TICRR,TGFB1,AURKA,CCNB1,CDK1,RFWD3,PLK3,PLK2,CDK2,NOP53,NDC80,E2F1,TNKS1BP1,MAD2L1,CASP2,DONSON,FOXO4,BRCA1,MSH2,GEN1,ZNF207, ZNF385A,GTSE1,CNOT9,MUS81,CDKN1A,TOPBP1,KNTC1,UBA52,PIDD1,RB1,CNOT6,PML,ZWINT,BUB1B,BUB3,RINT1, XPC,NBN,HMGA2,TP53,ARID3A,PRKDC,CARM1,PCBP4,BLM,RPA2,TP63,TRIAP1,CDC25C,TIPIN,BUB1,ATF2,PLK1,BAX, VPS4A,HUS1,TTK,CNOT7,PRCC,CNOT10,CNOT8,NABP1,PRMT1,NAE1

cell cycle G1/S phase transition (GO:0044843)	RRM2,MCM4,ID2,MYC,MCM3,CDC6,ORC6,ORC1,EIF4EBP1,TCIM,SKP2,PKD1,CDC45,MCM10,E2F8,PCNA,MCM6,DHFR, FBXO5,CDC25A,GADD45A,CCNA2,CDC7,KLF4,TFDP1,TYMS,MCM7,POLA2,MCM2,POLE,AURKA,CCNB1,EZH2,CDK1,FAM83D,RFWD3,PLK3,PLK2,CDK2,NASP,DACT1,MCM8,E2F1,TNKS1BP1,MCM5,CASP2,RBL1,CCNE2, ZNF385A,GTSE1,CNOT9,MTBP,CDKN1A,ORC5,WEE1,UBA52,PIDD1,RB1,BID,CNOT6,PML,POLE3,CTDSP1,CDKN3,ACVR1B,ATP2B4,CDC34,RPA1,ORC3, TP53,ARID3A,POLE2,CTDSPL,PRKDC,CARM1,PCBP4,ACVR1,RANBP1,DBF4,POLA1,ECD,RCC1,RPA2,TP63,GPNMB, CACUL1,TRIAP1,GLI1,RDX,CDC25C,TAF10,RBBP8,PPAT,UBE2E2,DDRGK1,PRIM2,PSME3,BAX,EIF4E,STXBP4,CTDSP2, CNOT7,CNOT10,CDK6,ANXA1,CNOT8,USP37,CDKN2D,PRMT1
nuclear DNA replication(GO:0033260)	FEN1,GINS2,GMNN,CDC45,GINS1,PCNA,RFC3,CDC7,BCL6,POLA2,RFC4,POLE,POLD4,SLBP,DONSON,DNA2,RFC2,RFC5, WDR18,FGFR1,LIG1,POLE3,RPA1,ORC3,POLE2,WIZ,POLA1,RPA2,TIPIN,PRIM2,POLD3,POLD1,DACH1
G1/S transition of mitotic cell cycle (GO:0000082)	RRM2,MCM4,ID2,MYC,MCM3,CDC6,ORC6,ORC1,EIF4EBP1,SKP2,PKD1,CDC45,MCM10,E2F8,PCNA,MCM6,DHFR,FBXO5, CDC25A,GADD45A,CDC7,KLF4,TFDP1,TYMS,MCM7,POLA2,MCM2,POLE,AURKA,CCNB1,EZH2,CDK1,RFWD3,PLK3,PLK2, CDK2,NASP,DACT1,MCM8,E2F1,TNKS1BP1,MCM5,CASP2,RBL1,CCNE2,ZNF385A,GTSE1,CNOT9,MTBP,CDKN1A,ORC5, WEE1,UBA52,PIDD1,RB1,BID,CNOT6,PML,POLE3,CTDSP1,CDKN3,ACVR1B,CDC34,RPA1,ORC3,TP53,ARID3A,POLE2, CTDSPL,PRKDC,CARM1,PCBP4,ACVR1,RANBP1,DBF4,POLA1,ECD,RCC1,RPA2,TP63,GPNMB,CACUL1,TRIAP1,RDX, CDC25C,TAF10,RBBP8,PPAT,UBE2E2,PRIM2,PSME3,BAX,EIF4E,CTDSP2,CNOT7,CNOT10,CDK6,ANXA1,CNOT8,USP37, CDKN2D,PRMT1

DNA biosynthetic process (GO:0071897)	MYC,DTL,PCLAF,CTGF,PCNA,GREM1,RFC3,XRCC2,EXO1,KLF4,TYMS,DUSP1,POLA2,RFC4,HSP90AA1,RAD51AP1,POLE, TK1,DSCC1,UFD1,POLD4,TNKS1BP1,PKIB,VCP,MAPK3,DNA2,BRCA1,RFC2,BARD1,XRCC5,SRC,NHP2,NEK7,RFC5, CDKN1A,BRIP1,UBA52,CCT2,CCT5,LIG1,PDGFRB,POLE3,PRKD2,SPRTN,MAP2K7,ARRB2,NBN,POLL,TCP1,RPA1,CCT8, CTC1,FAAP20,TP53,KAT5,POLE2,RMI1,RAD51C,POLA1,BLM,CTNNB1,RPA2,RMI2,RBBP8,CCT6A,PRIM2,UBE2L6,POLQ, SMG6,HSP90AB1,NOP10,POLD3,PNKP,FBXO4,NEK2,SMG5,POLM,POLD1,TENT4B,TRIM25,HNRNPA2B1,POLG,CDKN2D, DACH1,DDX39B
DNA integrity checkpoint (GO:0031570)	WDR76,CDC6,CDC45,DTL,E2F8,TIMELESS,PCNA,GADD45A,CLSPN,TFDP1,BRSK1,FANCI,TICRR,AURKA,CCNB1,CDK1, RFWD3,PLK3,PLK2,CDK2,NOP53,E2F1,TNKS1BP1,CASP2,DONSON,FOXO4,INTS7,DNA2,BRCA1,MSH2,ZNF385A,GTSE1, CNOT9,MUS81,CDKN1A,BRIP1,TOPBP1,UBA52,PIDD1,CNOT6,PML,RINT1,XPC,NBN,HMGA2,TP53,ARID3A,H2AFX,PRKDC,CARM1,PCBP4,BLM,RPA2,TP63,TRIAP1,CDC25C,TIPIN,RBBP8,ATF2,RAD1,PLK1,CDC14B,ABRAXAS1,BAX,HUS1,FBXO4,CNOT7,CHEK1,CNOT10,FZR1,CNOT8,ATRIP,TIPRL,DDX39B,PRMT1,NAE1
sister chromatid segregation (GO:0000819)	FEN1,CDC6,CENPK,SKA2,CENPI,ZWILCH,SKA1,TRIP13,CENPU,DSN1,SPC25,DYNC1I1,NCAPG,NCAPH,DUSP1,CENPO, RACGAP1,CCNB1,DSCC1,CHMP1B,ERCC6L,NCAPD3,KIF4A,CENPN,CENPM,NDC80,CDC23,MAD2L1,MCMBP,SPAG5,NUF2, SMC1A,CDCA5,KIF23,GEN1,PPP1CC,ZNF207,SLF1,KNTC1,SGO1,KIF2C,RB1,NUP85,UBE2C,SPDL1,TACC3,ZWINT, NUP107,BUB1B,KNL1,BUB3,CDCA8,CENPQ,NSL1,KPNB1,DLGAP5,NUP43,CENPL,CENPP,CHTF8,AKAP8L,RAD51C,SMC2, SMC4,CTNNB1,AURKC,CEP57L1,RAN,RMI2,SFPQ,CENPH,NUMA1,MAPRE1,H2AFY,BUB1,KNSTRN,CHMP2A,

	ANAPC5,SPC24, CENPE,KIF22,HECW2,PLK1,CDC14B,NCAPD2,XPO1,VPS4A,PHF23,KIFC1,TTK,NEK2,NUP160,CTCF,MIS12,PTTG1, DYNC1I2,KIF18B,DYNLL1
mitotic DNA damage checkpoint (GO:0044773)	E2F8,PCNA,GADD45A,CLSPN,TFDP1,BRSK1,FANCI,AURKA,CCNB1,CDK1,RFWD3,PLK3,PLK2,CDK2,NOP53,E2F1, TNKS1BP1,CASP2,DONSON,FOXO4,MSH2,ZNF385A,GTSE1,CNOT9,MUS81,CDKN1A,TOPBP1,UBA52,PIDD1,CNOT6,PML, RINT1,XPC,NBN,HMGA2,TP53,ARID3A,PRKDC,CARM1,PCBP4,BLM,RPA2,TP63,TRIAP1,CDC25C,TIPIN,ATF2, BAX,HUS1,CNOT7,CNOT10,CNOT8,PRMT1
mitotic DNA integrity checkpoint (GO:0044774)	E2F8,PCNA,GADD45A,CLSPN,TFDP1,BRSK1,FANCI,TICRR,AURKA,CCNB1,CDK1,RFWD3,PLK3,PLK2,CDK2,NOP53,E2F1, TNKS1BP1,CASP2,DONSON,FOXO4,MSH2,ZNF385A,GTSE1,CNOT9,MUS81,CDKN1A,TOPBP1,UBA52,PIDD1,CNOT6,PML, RINT1,XPC,NBN,HMGA2,TP53,ARID3A,PRKDC,CARM1,PCBP4,BLM,RPA2,TP63,TRIAP1,CDC25C,TIPIN,ATF2, BAX,HUS1,CNOT7,CNOT10,CNOT8,PRMT1,NAE1
cell cycle DNA replication(GO:0044786)	FEN1,GINS2,GMNN,CDC45,E2F8,GINS1,PCNA,RFC3,CDC7,BCL6,POLA2,RFC4,POLE,POLD4,SLBP,DONSON,DNA2,RFC2, SMC1A,RFC5,WDR18,FGFR1,LIG1,POLE3,RPA1,ORC3,POLE2,WIZ,POLA1,RPA2,TIPIN,PRIM2,POLD3,POLD1,DACH1

negative regulation of cell cycle G2/M phase transition (GO:1902750)	DTL,CLSPN,PSMC2,BRSK1,FANCI,TICRR,AURKA,PSMD14,CDK1,PSMD12,NOP53,PSMD2,DONSON,FOXO4,PSMB10,BRCA1, PSMD1,PSMA4,PSMD11,MUS81,TOPBP1,UBA52,PSMD6,SKP1,PSMA1,MIIP,PSME4,RINT1,NBN,HMGA2,PSMD7,RBX1, PSMA3,PSMD5,PSMB1,BLM,H2AFY,PLK1,CDC14B,PSME3,ABRAXAS1,VPS4A,PSMC5,HUS1,PSMA2,PSMD10, CHEK1,FZR1, PSMA5,PSMC6,NAE1
signal transduction involved in cell cycle checkpoint(GO:0072395)	DTL,E2F8,PCNA,GADD45A,TFDP1,AURKA,CCNB1,CDK1,PLK3,PLK2,CDK2,E2F1,TNKS1BP1,CASP2,BRCA1,ZNF385A, GTSE1,CNOT9,CDKN1A,UBA52,PIDD1,CNOT6,PML,RINT1,TP53,ARID3A,PRKDC,CARM1,PCBP4,TRIAP1,CDC25C,PLK1, CDC14B,ABRAXAS1,BAX,CNOT7,CHEK1,CNOT10,FZR1,CNOT8,PRMT1
signal transduction involved in DNA integrity checkpoint(GO:0072401)	DTL,E2F8,PCNA,GADD45A,TFDP1,AURKA,CCNB1,CDK1,PLK3,PLK2,CDK2,E2F1,TNKS1BP1,CASP2,BRCA1,ZNF385A, GTSE1,CNOT9,CDKN1A,UBA52,PIDD1,CNOT6,PML,RINT1,TP53,ARID3A,PRKDC,CARM1,PCBP4,TRIAP1,CDC25C,PLK1, CDC14B,ABRAXAS1,BAX,CNOT7,CHEK1,CNOT10,FZR1,CNOT8,PRMT1
signal transduction involved in DNA damage checkpoint(GO:0072422)	DTL,E2F8,PCNA,GADD45A,TFDP1,AURKA,CCNB1,CDK1,PLK3,PLK2,CDK2,E2F1,TNKS1BP1,CASP2,BRCA1,ZNF385A, GTSE1,CNOT9,CDKN1A,UBA52,PIDD1,CNOT6,PML,RINT1,TP53,ARID3A,PRKDC,CARM1,PCBP4,TRIAP1,CDC25C,PLK1, CDC14B,ABRAXAS1,BAX,CNOT7,CHEK1,CNOT10,FZR1,CNOT8,PRMT1

chromosome segregation (GO:0007059)	FEN1,CDC6,CENPK,SKA2,CENPI,ZWILCH,SKA1,TRIP13,CENPU,DSN1,SPC25,DYNC1I1,NCAPG,NCAPH,ESCO2,DUSP1, CENPO,RACGAP1,CSNK2A2,CCNB1,DSCC1,FAM83D,CHMP1B,ERCC6L,NCAPD3,CENPW,KIF4A,CENPN,CENPM,NDC80,CDC23,MAD2L1,CIAO2B,MCMBP,BRCA1,SPAG5,NUF2,CCNE2,FANCD2,SMC1A,CDCA5,NDC1,KIF23,TOP1MT,ARL8A,GEN1,PPP1CC,ZNF207,MUS81,SLF1,BRIP1,KNTC1,SGO1,SKA3,KIF2C,ERCC2,RB1,NUP85,MKI67,UBE2C,SPDL1,TACC3,ZWINT, NUP107,BUB1B,KNL1,BUB3,TTL,CDCA8,CENPQ,HJURP,NSL1,KPNB1,DLGAP5,NUP43,CENPL,CENPP,CHTF8, RIOK3, AKAP8L,RAD51C,GEM,OIP5,SMC2,SMC4,CTNNB1,AURKC,CEP57L1,RAN,KIF11,RCC1,RMI2,SFPQ,CENPH,NUMA1,RAD18, MAPRE1,H2AFY,BUB1,KNSTRN,CHMP2A,P3H4,ACTR2,ANAPC5,SPC24,CENPE,KIF22,HECW2,PLK1,CDC14B, NCAPD2,TOP1,XPO1,VPS4A,PHF23,LEMD2,KIFC1,TTK,NEK2,NAA60,NUP160,RPS3,CTCF,MIS12,PTTG1,ARL8B,DYNC1I2,PPP1R7, KIF18B,DYNLL1

DEGs: differentially expressive genes.
