# Supplementary material for: Identification of ubiquitin-specific protease 32 as an oncogene in glioblastoma and the underlying mechanisms
Source: Sci Rep. 2022 Apr 19;12:6445. doi: 10.1038/s41598-022-09497-y (PMC9018837; doi:10.1038/s41598-022-09497-y)
Supplement: Supplementary file 3 — Supplementary Table S2. [file 41598_2022_9497_MOESM3_ESM.pdf]

Table S2. Kyoto Encyclopedia of Genes and Genomic pathway (KEGG) enrichment analysis

KEGG pathway	Number of DEGs involved in pathway	% of total DEGs involved in pathway	P-value	DEGs
Cell cycle	67	4.22	6.8832×10^{-11}	MCM4,MYC,MCM3,CDC6,ORC6,E2F2,ORC1,SKP2,CDC45,PCNA,MCM6,CDC25A,GADD45A,CCNA2,CDC7,TFDP1,YWHAH,MCM7,MCM2,HDAC1,TGFB1,CCNB1,CDK1,CDK2,CDC23, E2F1,MAD2L1,MCM5,RBL1,CCNE2,SMC1A,TGFB2,CDKN1A,ORC5,WEE1,SKP1,RB1, BUB1B,BUB3,YWHAG,ORC3,PKMYT1,TP53,RBX1,PRKDC,ABL1,DBF4,GADD45B,CDC25C,BUB1,ANAPC5,PLK1,CDC14B,YWHAZ,YWHAB,CCNB2,ANAPC7,YWHAQ,E2F5,TTK,CHEK1,CDK6,PTTG1,FZR1,CDC16,CDKN2D,ANAPC13
DNA replication	25	1.58	1.9050×10^{-7}	MCM4,FEN1,MCM3,PCNA,MCM6,RFC3,MCM7,POLA2,RFC4,MCM2,POLE,POLD4,MCM5, DNA2,RFC2,RFC5,LIG1,POLE3,RPA1,POLE2,POLA1, RPA2,PRIM2,POLD3,POLD1
Human T-cell leukemia virus 1 infection	79	4.98	0.0007	EGR1,MYC,MYBL2,WNT7B,E2F2,RELB,PCNA,IL6,NFATC4,PDGFA,BCL2L1,RRAS, NFKB2,ATF4,SLC25A4,ICAM1,DVL2,APC2,POLE,TGFB1,SLC25A6,JAK3,SRF,VDAC3, POLD4,CDC23,E2F1,MAD2L1,ZFP36,TGFB2,DVL1,CANX,CDKN1A,RB1,TGFBR2, PDGFRB,RELA,POLE3,BUB1B,CRTC1,ADCY8,BUB3,PIK3CD,CRTC2,MAPK8,FZD8,TP53,KAT5,POLE2,NFATC2,TCF3,RANBP1,FZD2,CREM,CTNNB1,RAN,CALR,CHUK,ATF2, NFKBIA,ANAPC5,EGR2,MAP3K1,FZD7,XPO1,BAX,CCNB2,POLD3,ANAPC7,LTBR,IL1R1,FZD1,ATF3,TSPO,CHEK1,PTTG1,ELK1,POLD1,CDC16
Base excision repair	18	1.13	0.0016	FEN1,PCNA,POLE,POLD4,NEIL3,LIG1,PARP1,POLE3,POLL,HMGB1,POLE2,MPG, PARP3,TDG,XRCC1,POLD3,MBD4,POLD1
Mismatch repair	13	0.82	0.0028	PCNA,RFC3,EXO1,RFC4,POLD4,RFC2,MSH2,RFC5,LIG1,RPA1,RPA2,POLD3,POLD1

Cellular senescence	55	3.47	0.0035	MYC,MYBL2,CXCL8,E2F2,EIF4EBP1,IL6,NFATC4,CDC25A,GADD45A,CCNA2,RRAS, SLC25A4,SQSTM1,CALM1,TGFB1,CCNB1,CDK1,SLC25A6,VDAC3,MAP2K2,CDK2,E2F1, SERPINE1,RBL1,MAPK3,CCNE2,TGFB2,PPP1CC,CDKN1A,RB1,TGFBR2,RELA,RBBP4, PIK3CD,CALM3,HIPK3,NBN,TP53,NFATC2,LIN54,ZFP36L1,GADD45B,LIN52,CAPN2, RAD1,MAP2K3,TRAF3IP2,MAPK12,HIPK2,CCNB2,HUS1,E2F5,CHEK1,CDK6,PPP1CB
Hepatitis B	49	3.09	0.0040	MYC,CXCL8,E2F2,PCNA,IL6,NFATC4,CCNA2,ATF4,CREB3L1,TGFB1,VDAC3,MAP2K2, CDK2,IRF7,E2F1,MAPK3,CCNE2,SRC,TGFB2,CDKN1A,TICAM1,RB1,RELA,PIK3CD, MAPK8,CYCS,TP53,NFATC2,HSPG2,CHUK,ATF2,NFKBIA,CREB3L4,MAPK9,EGR2, MAP3K1,IKBKE,YWHAZ,CREB3L2,YWHAB,BAD,BAX,YWHAQ,TBK1,CDK6,ELK1,DDX58, STAT2,TLR3
Proteasome	19	1.20	0.0063	PSMC2,PSMD14,PSMD12,PSMD2,PSMB10,PSMD1,PSMA4,PSMD11,PSMD6,PSMA1,PSME4,PSMD7,PSMA3,PS MB1,PSME3,PSMC5,PSMA2, PSMA5,PSMC6
Epithelial cell signaling in Helicobacter pylori infection	27	1.70	0.0067	ATP6V1F,CXCL8,ATP6V1B2,JAM2,SRC,ATP6V1G1,ATP6V0E1,TCIRG1,F11R,ATP6V1A,RELA,ATP6V1C1,PAK 1,MAPK8,ATP6V1E1,ATP6V0C,GIT1,CHUK,ATP6V0D1,NFKBIA, MAPK9,JAM3,ATP6V1H, MAPK12,ATP6V1C2,ATP6V1D,TJP1
Nucleotide excision repair	20	1.26	0.0071	PCNA,RFC3,RFC4,POLE,GTF2H1,POLD4,RFC2,RFC5,ERCC2,RAD23A,LIG1,POLE3, XPC,RPA1,POLE2,RBX1,RPA2,ERCC1,POLD3,POLD1
Synaptic vesicle cycle	22	1.39	0.0085	ATP6V1F,STXBP1,ATP6V1B2,VAMP2,ATP6V1G1,ATP6V0E1,TCIRG1,STX3,ATP6V1A, ATP6V1C1,ATP6V1E1,CLTCL1,ATP6V0C,ATP6V0D1,STX1B,STX2,SNAP25,NAPA, ATP6V1H,ATP6V1C2,ATP6V1D,AP2A1
Collecting duct acid secretion	12	0.76	0.0096	ATP6V1F,ATP6V1B2,ATP6V1G1,ATP6V0E1,TCIRG1,ATP6V1A,ATP6V1C1,ATP6V1E1, ATP6V0C,ATP6V0D1,ATP6V1C2,ATP6V1D

Oocyte meiosis	43	2.71	0.0097	FBXO5,RPS6KA2,YWHAH,CALM1,AURKA,CCNB1,CDK1,CDK2,CDC23,MAD2L1,AR,MAPK3,CCNE2,SMC1A,PPP1CC,PPP2CA,SGO1,SKP1,ADCY8,YWHAG,CALM3,PKMYT1,RBX1,PPP2R5A,CDC25C,BUB1,PPP2R1B,ANAPC5,FBXO43,PLK1,YWHAZ,PPP2CB,YWHAB,MAPK12,PPP2R5B,CCNB2,ANAPC7,YWHAQ,PTTG1,IGF1R,CDC16,PPP1CB,ANAPC13
Protein processing in endoplasmic reticulum	56	3.53	0.0130	HSP90B1,DNAJA1,ATF4,PDIA4,HSP90AA1,DNAJB11,HSPA8,TRAM1,HSPA5,DNAJC3,UFD1,ERN1,VCP,BAG1,SVIP,HSPH1,PPP1R15A,RPN2,ERP29,CANX,DDIT3,SKP1,UBXN6,RAD23A,YOD1,STT3B,MAPK8,MAP2K7,UBQLN4,SEC24D,SSR3,RBX1,PRKCSH,SSR4,EIF2S1,DERL1,UGGT1,SEC61G,EIF2AK3,MOGS,PLAA,CALR,CAPN2,MAPK9,SAR1B,SSR1,DERL2,BAX,HSP90AB1,STUB1,SEC23B,SEC23A,RNF185,HYOU1,UBE2D1,NGLY1
Steroid biosynthesis	10	0.63	0.0152	TM7SF2,DHCR7,SQLE,CYP51A1,LIPA,SOAT1,CYP27B1,HSD17B7,FDFT1,MSMO1
FoxO signaling pathway	45	2.84	0.0163	PCK2,SKP2,IL6,GADD45A,SOD2,IRS2,BCL6,TGFB1,CCNB1,PLK3,MAP2K2,PLK2,CDK2,STK11,PLK4,KLF2,FOXO4,CSNK1E,MAPK3,TGFB2,PRKAB1,CDKN1A,TGFBR2,PIK3CD,HOMER1,MAPK8,SGK3,CAT,FBXO32,AGAP2,G6PC3,GADD45B,CHUK,MAPK9,IRS1,SETD7,PLK1,MAPK12,GABARAPL1,CCNB2,PDPK1,IGF1R,CDKN2D,INSR,PRMT1

PI3K-Akt signaling pathway	93	5.86	0.0209	DDIT4,PCK2,MYC,EREG,CSF3,IL6R,GNG2,EIF4EBP1,IL6,ERBB2,PDGFA,BCL2L1, HSP90B1,ATF4,ITGA7,CREB3L1,YWHAH,NR4A1,HSP90AA1,CSF1,JAK3,MAP2K2,CDK2,STK11,THBS4,VEGFB,MAPK3,BRCA1,ITGA11,CCNE2,MLST8,FGF5,COL9A2,PPP2CA, CDKN1A,THBS2,COL4A2,GNB2,VEGFA,FGFR1,EIF4B,PDGFRB,RELA,EPOR,PIK3CD, PRLR,YWHAG,CRTC2,PKN1,COL6A2,GNG10,TP53,SGK3,CDC37,BDNF,SPP1,PPP2R5A, LAMA5,COL4A5,G6PC3,THEM4,CHUK,ATF2,CREB3L4,PPP2R1B,IRS1,THBS3,YWHAZ, CREB3L2,PPP2CB, PTK2,GNG5,YWHAB,BAD,NGFR,EIF4E,COL1A1,HSP90AB1,PPP2R5B, YWHAQ,PDPK1,FGF1,PKN3,CDK6,ITGA3,EFNA3,ITGB5,PHLPP2,IGF1R,MTCP1,INSR, GNG11,ITGA10
Epstein-Barr virus infection	54	3.40	0.0233	MYC,E2F2,RELB,SKP2,IL6,OAS1,GADD45A,CCNA2,PSMC2,NFKB2,OAS3,ICAM1, HDAC1,PSMD14,JAK3,PSMD12,OAS2,CDK2,PSMD2,IRAK1,IRF7,E2F1,PSMD1,CCNE2, PSMD11,NFKBIE,CDKN1A,PSMD6,RB1,BID,RELA,PIK3CD,MAPK8,CYCS,MAP2K7, PSMD7,TP53,TNFAIP3,GADD45B,CALR,CHUK,NFKBIA,MAPK9,MAP2K3,IKBKE,MAPK12,BAX,PSMC5,TBK1 ,CDK6,DDX58,STAT2,TRAF5,PSMC6
TNF signaling pathway	37	2.33	0.0275	LIF,CXCL2,CXCL3,IL1B,IL6,IRF1,CEBPB,ATF4,MMP3,CREB3L1,JUNB,ICAM1, BIRC2,CSF1,MAPK3,BCL3,SOCS3,RELA,MMP14,PIK3CD,MAPK8,MAP2K7,DAB2IP, MAP3K8,TNFAIP3,PTGS2,CHUK,ATF2,NFKBIA,TNFRSF1B,CREB3L4,MAPK9,MAP2K3, CREB3L2,MAPK12,MLKL,TRAF5
Herpes simplex infection	49	3.09	0.0326	SKP2,IL1B,IL6,OAS1,SRSF1,OAS3,PER1,C3,CSNK2A2,CDK1,SRSF2,SRSF3,OAS2, CDK2,NOP53,IRF7,NECTIN2,PPP1CC,GTF2IRD1,TICAM1,SKP1,SOCS3,PML,RELA, SRSF7,MAPK8,CYCS,CDC34,IFIT1,TP53,EIF2S1,EIF2AK3,TAF13,HCFC2,TAF9B, CHUK,TAF10,NFKBIA,NXF3,MAPK9,IKBKE,SRSF6,TBK1,DDX58,STAT2,PPP1CB, TRAF5,TLR3,C1QBP

IL-17 signaling pathway	29	1.83	0.0331	CXCL2,CSF3,CXCL8,CXCL3,IL1B,IL6,JUND,SRSF1,CEBPB,HSP90B1,MMP3, HSP90AA1,FOSB,MAPK3,RELA,MAPK8,TNFAIP3,PTGS2,CHUK,NFKBIA,MAPK9,ANAPC5,IKBKE,TRAF3IP2, TRAF4,MAPK12,HSP90AB1,TBK1,TRAF5
Glycerophospholipid metabolism	32	2.02	0.0392	TAZ,DGKQ,LPCAT4,GPAT3,PLD3,CDS1,PCYT1B,AGPAT5,GPAM,PLD2,PCYT2,SELENOI,LPCAT1,PLA2G6,DGKG,LCLAT1,LYPLA1,PTDSS1,AGPAT4,LPCAT2,GPD2,PLPP4, AGPAT1,LCAT,DGKA,MBOAT2,DGKD,PNPLA6,DGKI,PTDSS2,GPAT4,PCYT1A
Pyruvate metabolism	15	0.95	0.0396	PCK2,PDHA1,LDHA,LDHB,FH,LDHD,ALDH3A2,ACSS2,GRHPR,ACAT1,MDH1,ACACB, HAGH,DLD,GLO1
Homologous recombination	17	1.07	0.0429	RAD54L,XRCC2,POLD4,BRCA1,BARD1,MUS81,BRIP1,TOPBP1,NBN,RPA1,RAD51C,BLM,RPA2,RBBP8,ABRAXAS1,POLD3,POLD1
Cholesterol metabolism	17	1.07	0.0429	LDLR,NCEH1,VDAC3,PLTP,ABCA1,OSBPL5,NPC2,NPC1,LIPA,SOAT1,ANGPTL4,VAPB, LCAT,STARD3,TSPO,SCARB1,SORT1
Hepatitis C	38	2.40	0.0499	CXCL8,LDLR,OAS1,IRF1,OAS3,OAS2,IRF7,MAPK3,PPP2CA,CDKN1A,TICAM1,SOCS3, NR1H3,RELA,PIK3CD,MAPK8,IFIT1,TP53,EIF2S1,EIF2AK3,CD81,CLDN7,CHUK, CLDN1,NFKBIA,PPP2R1B,MAPK9,IKBKE,PPP2CB,MAPK12,PSME3,BAD,PDPK1,TBK1, SCARB1,DDX58,STAT2,TLR3

DEGs: differentially expressive genes.
